# The DCBLD2 super-enhancer drives colorectal cancer progression through FOSL2/JUND-mediated activation of the CD146/AKT/TNFRSF6B pathway

**DOI:** 10.1186/s43556-026-00592-4

**Published:** 2026-09-23

**Authors:** Tong Wu, Lan Shao, Xin Hu, Hongjuan He, Lu Chen, Meiqi Feng, Chengjin Qi, Ziwen Wang, Haoran Yu, Boshu Ji, Yijia Cao, Yan Zhang, Qiong Wu

**Affiliations:** 1 School of Life Science and Technology, Faculty of Life Sciences and Medicine, Harbin Institute of Technology, Harbin, 150001 China; 2 Department of Medicine, Dalian University of Technology, Dalian, China; 3 Department of Pathology, the Second Affiliated Hospital of Harbin Medical University, Harbin, China; 4 Department of Biomedical Engineering, School of Medical Information and Engineering, Guangdong Pharmaceutical University, Guangzhou, China

**Keywords:** Colorectal cancer, Super-enhancer, Activator protein-1 transcription factors, DCBLD2, AKT signaling pathway, Epigenetic regulation

## Abstract

Super-enhancers are clusters of cis-regulatory elements that sustain the expression of cell-identity genes. Their aberrant activation or dysfunction constitutes a hallmark of malignant cellular transformation, which frequently rewires oncogenic transcriptional programs to support uncontrolled cell proliferation and metastatic potential. However, the functions and epigenetic regulation mechanisms of colorectal cancer specific super-enhancers remain poorly understood, and few studies have focused on dissecting how tumor enriched super-enhancers mediate downstream oncogenic signaling in CRC. By integrating multi-omics sequencing data, we identified a highly active super-enhancer in colorectal cancer, termed DCBLD2-SE. Through luciferase reporter assays, the SE3-2 subregion was identified as the minimal functional core of this super-enhancer. Mechanistic studies confirmed that transcription factors of the activator protein-1 family (FOSL2 and JUND) can bind directly to the SE3-2 cis-regulatory element, synergistically regulating local chromatin remodeling and strongly driving the upregulation of the target gene *DCBLD2*. Following knockout of the DCBLD2-SE core region, the malignant proliferative capacity of colorectal cancer cells decreased significantly, and the growth of xenograft tumors in vivo was markedly inhibited. Functionally, DCBLD2-SE facilitates tumor progression through a DCBLD2-dependent CD146/AKT/TNFRSF6B signaling axis. Clinically, high expression of *DCBLD2* correlates with malignant metastasis and poor overall survival in patients with colorectal cancer. In summary, this study uncovers a previously undescribed DCBLD2-centered regulatory cascade that promotes colorectal cancer initiation and progression. DCBLD2-SE and its binding transcription factor activator protein-1 represent promising prognostic biomarkers and actionable therapeutic targets for colorectal cancer.

**Supplementary Information:**

The online version contains supplementary material available at https://doi.org/10.1186/s43556-026-00592-4.

## Introduction

Colorectal cancer (CRC) is among the most prevalent malignancies of the digestive system worldwide. In recent years, both the incidence and mortality rates of its have continued to rise, rendering CRC the third most common cancer globally [1]. Current clinical management includes surgical resection, chemotherapy and targeted agents therapy; nevertheless, a substantial proportion of patients are diagnosed at locally advanced or metastatic stages and lose access to curative surgical options [2, 3]. Therefore, dissecting epigenetic regulatory mechanisms that drive CRC progression and identifying actionable diagnostic biomarkers and therapeutic targets remain an urgent unmet clinical need [4, 5].

Super-enhancers (SEs) are large clusters of cis-regulatory elements enriched for histone marks such as H3K27ac and H3K4me1, as well as high transcription-factors (TF) occupancy [6]. Unlike conventional enhancers, SEs exhibit substantially stronger transcriptional activation potential [7]. Tumorigenesis is typically accompanied by epigenetic remodeling of the SEs landscape [8]. Aberrantly activated SEs drive oncogene overexpression to sustain malignant cellular phenotypes [9, 10]. Pan-cancer epigenomic studies have identified tumor-specific SE signatures in various solid tumors, and SE-targeted intervention has emerged as a promising therapeutic avenue in oncology [11, 12]. Although BET-bromodomain inhibitors that disrupt SEs activity have shown preclinical antitumor efficacy, existing investigations largely focus on pan-cancer SEs profiling and upstream transcriptional regulation [13]. However, in-depth dissection of the regulatory interplay between CRC-associated SE cis-elements and their downstream transmembrane oncogenic receptors remains scarce. Therefore, characterizing tumor-specific SEs circuits may finform precision therapeutic strategies and mitigate off-target toxicity associated with broad-spectrum BRD4 inhibitors.

DCBLD2 is a conserved transmembrane receptor encoded at the human chromosome (chr) 3q12.1 locus, and its protein harbors CUB, LCCL, and coagulation factor V/VIII-homologous domains [14]. DCBLD2 modulates angiogenesis via platelet-derived growth factor and vascular endothelial growth factor signaling and contributes to tumor cell proliferation and invasion [15, 16]. Multi-omics analyses of data from the CPTAC cohorts confirmed that DCBLD2 is significantly overexpressed in CRC tissues and is an independent prognostic risk factor for CRC [17, 18]. Oncogenic functions of DCBLD2 have been reported across multiple cancer types; however, the epigenetic mechanisms underlying its pathological overexpression in CRC remain poorly systematically characterized.

In this work, we identified DCBLD2-SE as a previously uncharacterized, highly active SE in CRC that governs DCBLD2 transcription. We defined SE3-2 as its minimal functional core element. We further demonstrated that the AP-1 (activator protein-1) TFs FOSL2 and JUND cooperatively occupy this element to promote local chromatin activation. By combining multi-omics profiling, CRISPR/Cas9 genetic perturbation and in vitro as well as in vivo functional assays, we delineate the FOSL2/JUND-DCBLD2-SE-*DCBLD2* regulatory cascade. This epigenetic axis initiates downstream CD146/AKT/TNFRSF6B oncogenic signaling. Genetic ablation of SE3-2 dampens DCBLD2 expression, attenuates malignant cellular phenotypes and suppresses colorectal xenograft tumor growth. Our findings uncover an enhancer-driven oncogenic mechanism underlying CRC pathogenesis. Collectively, these results suggest that DCBLD2-SE and its associated transcriptional regulators could serve as candidate prognostic biomarkers and selective therapeutic targets.

## Results

### A highly active SE was screened and identified in the CRC cell lines

**Fig. 1:**
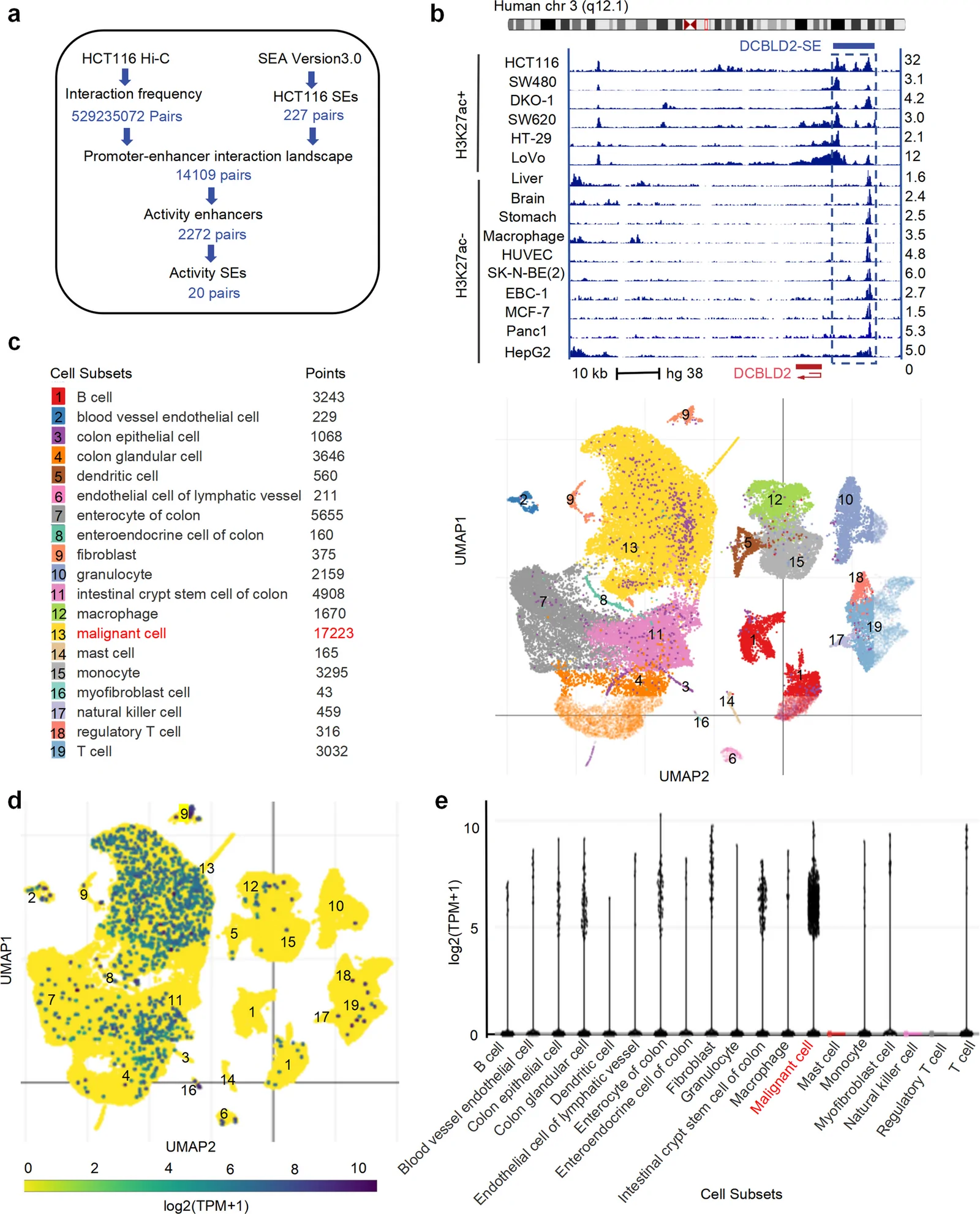
Identification and characterization of SEs in human CRC cells.** a** Genome-wide identification of SE sites with histone marks in HCT116 cells using the SEA (http://sea.edbc.org/) and 4DGenome Hi-C database (4dgenome.research.chop.edu). **b** ChIP-seq binding profiles showing H3K27ac enrichment at the q12.1 locus at the chr 3 q12.1 locus across CRC cell lines, normal tissues, immune cells, and other tumor cell lines. **c** The t-SNE cluster plot based on single-cell RNA-seq data (https://singlecell.broadinstitute.org/single_cell) from human primary CRC tumor tissues. Distinct cell subpopulations are color-coded, with each dot represents an individual cell. **d** UMAP feature plot showing the log₂(TPM + 1) expression intensity of DCBLD2 across all single cells, with different colors representing different expression levels. **e** Violin plot displaying *DCBLD2* log₂(TPM + 1) expression levels stratified by annotated cell subsets*.* Single-Cell Portal (https://singlecell.broadinstitute.org/single_cell) provided the single-cell RNA-seq data and the associated annotation metadata

The identification of CRC-associated SEs is critical for understanding tumour epigenetic regulation. We therefore mined the SEA database and identified 227 cis-regulatory elements in HCT116 cells (Fig. 1a and Table S1). We next intersected these SE candidates with publicly available HCT116 Hi-C data. Based on chromatin interaction profiles, we identified 20 candidate SE loci marked by H3K27ac modifications and robust enhancer-promoter contacts. Among the top 10 SE target genes ranked by SE activity, only *DCBLD2* was highly expressed in CRC (Tables S2 and S3). Given this expression feature, we named the chr 3 located, highly active SE from HCT116 cells DCBLD2-SE for subsequent functional studies. We retrieved H3K27ac ChIP-seq data across 16 cell types and tissues and examined signal profiles visually to evaluate whether DCBLD2-SE activity showed greater enrichment in CRC (Fig. 1b). Substantial H3K27ac enrichment was detected at this SE locus across six CRC cell lines (HCT116, SW480, DKO-1, SW620, HT-29 and LoVo), with HCT116 exhibiting the most prominent signal. In contrast, far weaker H3K27ac signals were detected in normal tissues (liver, brain and stomach), non-tumor cell types (macrophages and HUVECs), and non-CRC tumor cell line (SK-N-BE(2), EBC-1, MCF-7, Panc1 and HepG2). Collectively, these findings prompted us, to further dissect the functional roles of DCBLD2-SE in CRC.

**Fig. 2:**
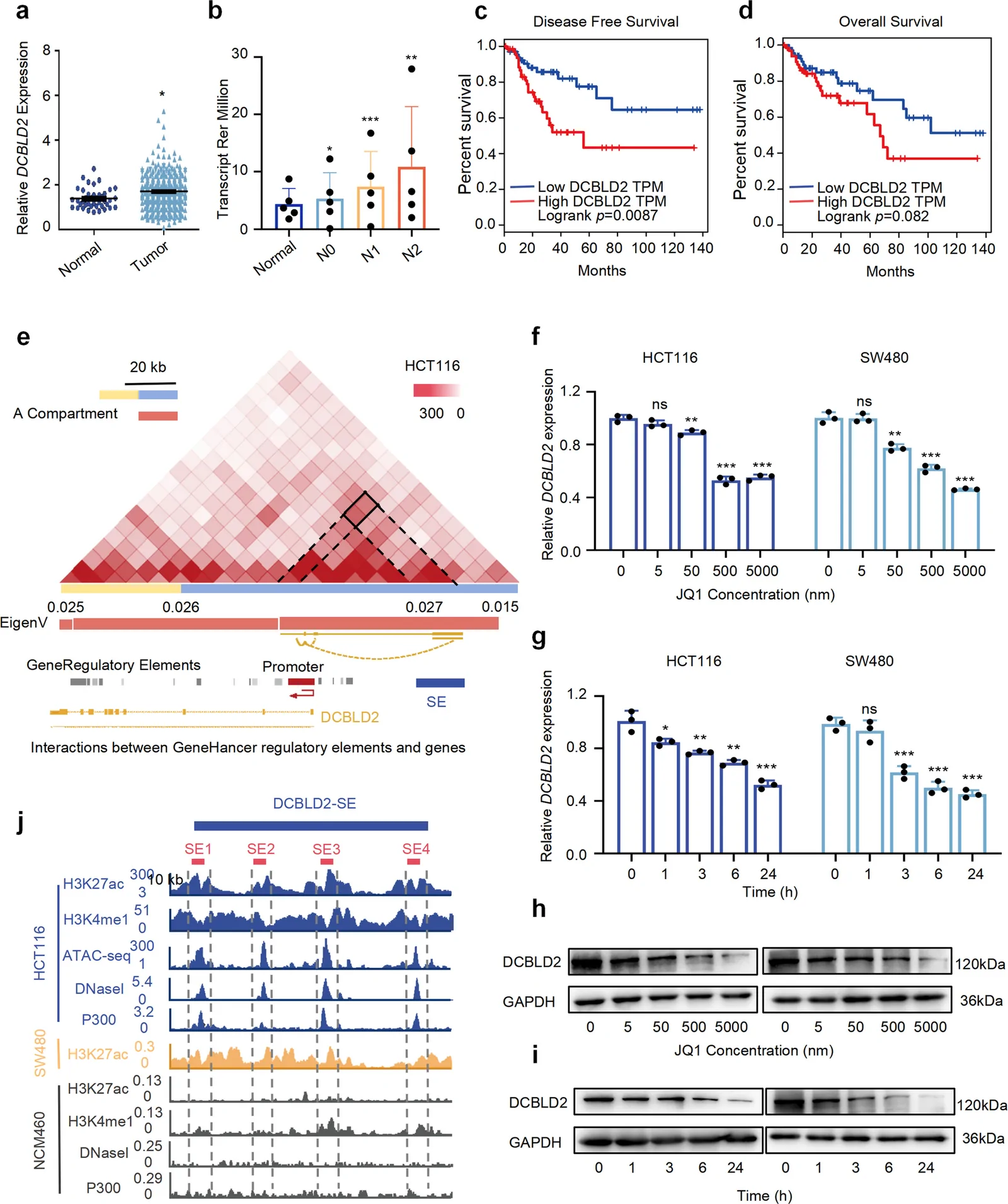
Elevated expression of DCBLD2 facilitates the progression and exacerbation of CRC.** a** *DCBLD2* expression levels in normal colon tissues (*n* = 41) and primary CRC tumors (*n* = 286) analyzed via the UALCAN database (ualcan.path.uab.edu). **b** *DCBLD2* expression across CRC subgroups with different lymph-node metastatic status using UALCAN (Normal, regional lymph node metastasis, *n* = 41; N1, metastases in 1–3 axillary lymph nodes, *n* = 166; N2, metastases in 4–9 regional lymph nodes, *n* = 70; N3, metastases in ≥ 10 axillary lymph nodes, *n* = 47). **c **and** d** Kaplan–Meier curves for disease-free survival and overall survival stratified by DCBLD2 expression in CRC patients (*n* = 68), generated from the GEPIA2 database (http://gepia2.cancer-pku.cn). The log-rank test was used for group-wise comparison. **e** Hi-C interaction heatmap of TAD chromatin loops surrounding the SE region in HCT116 cells; color intensity represents chromatin interaction frequency (4dgenome.research.chop.edu). **f** HCT116 and SW480 were treated with different concentrations of the BRD4 inhibitor JQ1 (0–5000 nM) for 24 h. The relative expression levels of DCBLD2 mRNA in cells were determined using RT-qPCR analysis (*n* = 3). *GAPDH* was used as an internal control. **g** HCT116 and SW480 cells were treated with 500 nM JQ1 for 0, 1, 3, 6, and 24 h, followed by RT-qPCR analysis to determine the mRNA expression levels of DCBLD2. The expression of *GAPDH* was used to standardize the RT-qPCR results (*n* = 3). **h** Western blot analysis of DCBLD2 protein expression levels in HCT116 and SW480 cells after 24 h treatment with 0–5000 nM JQ1. **i** Western blot analysis of DCBLD2 protein expression levels in HCT116 and SW480 cells after treatment with 500 nM JQ1 for 0–24 h. **j** Multi-omics epigenetic tracks (H3K27ac, H3K4me1, ATAC-seq, DNase I and P300) at the DCBLD2 locus in HCT116, SW480 and normal colon epithelial NCM460 cells, identifying four discrete constituent enhancer fragments (SE1, SE2, SE3 and SE4) of DCBLD2-SE. **f** and **g** Three biological replicates’ means are displayed. SEMs are indicated by error bars. With a two-tailed Student’s t test, *P < 0.05, **P < 0.01, ***P < 0.001, and ns, not significant

To explore the clinical significance of *DCBLD2*, we analyzed single-cell RNA-seq datasets containing 48,419 individual CRC cells (Fig. 1c). A total of 19 major cell subsets were identified, with malignant epithelial cells accounting for the largest proportion, totaling 17,223 cells. All cell subpopulation annotations demonstrated prominent enrichment of DCBLD2 transcripts within malignant epithelial tumor cell clusters (Fig. 1d, e). UALCAN database analysis further confirmed that the expression of *DCBLD2* was significantly higher in CRC tumor tissues relative to normal colon samples (Fig. 2a). Moreover, *DCBLD2* transcript levels also increased progressively with advancing lymphatic metastasis stage in CRC patients (Fig. 2b). Survival analysis using the GEPIA2 database showed that elevated *DCBLD2* mRNA was correlated with overall survival and disease-free survival in the CRC patient cohort (Fig. 2c, d). Collectively, single-cell and clinical dataset analyses indicate that high *DCBLD2* expression correlates with malignant progression and adverse clinical prognosis, supporting its potential as a prognostic biomarker for CRC.

### SE3-2 is the main active component of DCBLD2-SE

**Fig. 3:**
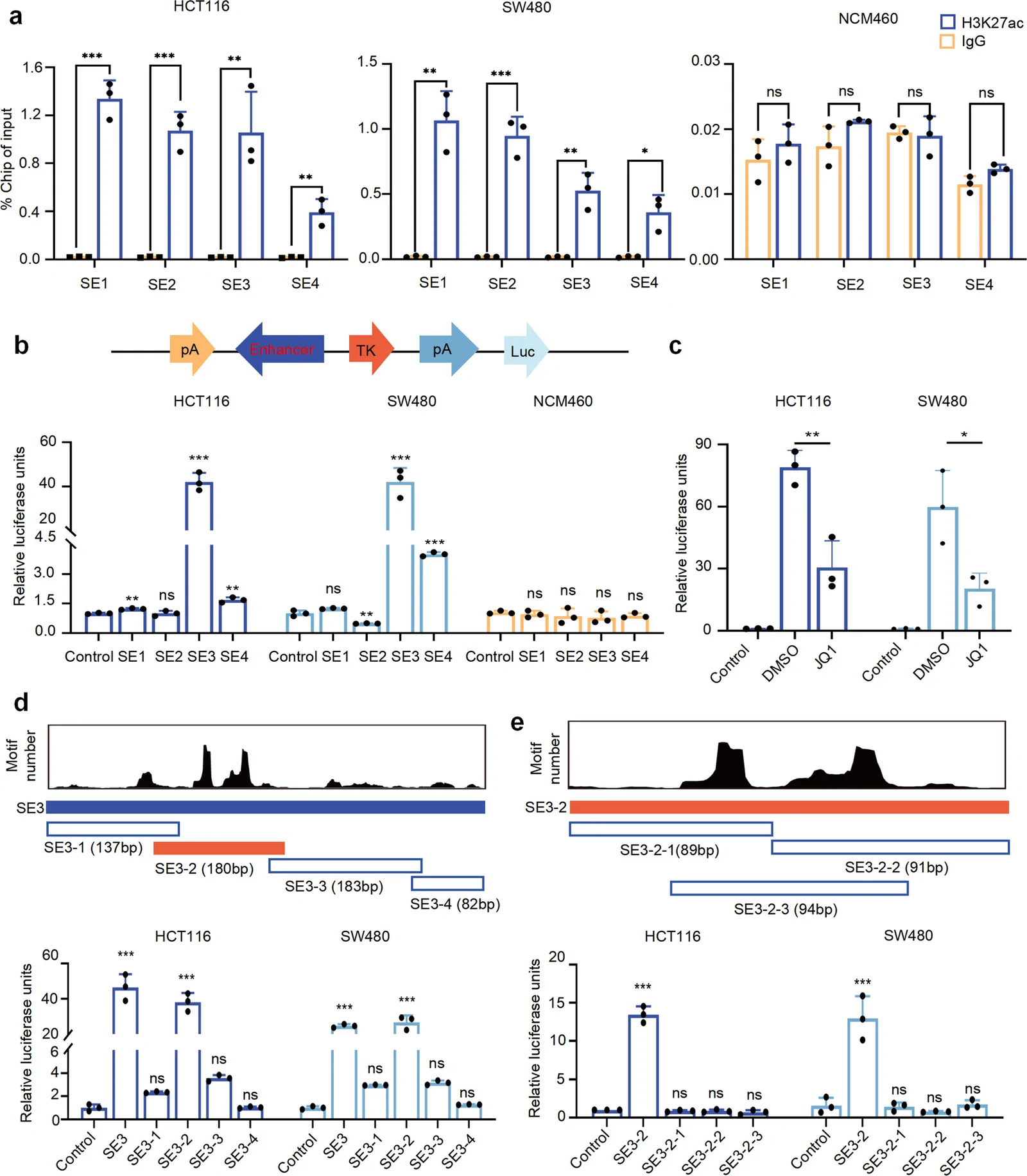
SE3-2 is the core functional fragment that drives DCBLD2-SE transcription.** a** ChIP-qPCR quantification of H3K27ac occupancy at SE1-SE4 in HCT116, SW480 and NCM460 (*n* = 3). Bar graphs display signal normalized to input chromatin. **b** Dual-luciferase reporter assay measuring intrinsic enhancer activity of four DCBLD2-SE fragments (SE1-SE4) in HCT116, SW480 and NCM460 cells (*n* = 3). **c** Assessment of SE3 luciferase activity following treatment of HCT116 and SW480 with 500 nM JQ1; DMSO served as the vehicle control (*n* = 3). **d** Dual-luciferase reporter assay showing that SE3-2 is the fragment of DCBLD2-SE that confers the predominant enhancer activity in HCT116 and SW480 cells (*n* = 3). **e** Dual-luciferase reporter assay to mapping the minimal active unit in the SE3-2 fragment in SW480 and HCT116 (*n* = 3). **b-e** PGL4.10 empty vector as a control. **a-e** Three biological replicates’ means are displayed. SEMs are indicated by error bars. With a two-tailed Student’s t test, *P < 0.05, **P < 0.01, ***P < 0.001, and ns, not significant

The genomic organization and functional regulatory properties of the newly identified DCBLD2-SE locus were next investigated through chromatin architecture analysis and pharmacological perturbation assays. Analysis of HCT116 Hi-C datasets revealed that the DCBLD2 promoter is 57 kb away from DCBLD2-SE within a shared topologically associating domain (TAD). The chromatin interaction profile results showed that this locus is located in the transcriptionally permissive A compartment and exhibits strong chromatin contacts within the TAD (Fig. 2e). BET-bromodomain inhibition mediated by JQ1, a well-characterized small-molecule inhibitor that disrupts SEs-driven transcription [11], was applied to examine whether DCBLD2 expression conforms to canonical SEs regulatory mechanisms. HCT116 and SW480 cells were treated with serially diluted JQ1 for 24 h. At 500 nM JQ1 treatment, both DCBLD2 mRNA and protein abundance were markedly reduced in HCT116 and SW480 cells (Fig. 2f-i and Fig. S1a, b). By contrast, JQ1 exposure at the same concentration did not substantially alter transcript levels of housekeeping control genes *ALB* and *GUSB* (Fig. S1c). These findings support that DCBLD2-SE transcription in HCT116 and SW480 cells is BRD4-dependent, consistent with canonical SEs regulation*.* Based on the characteristics of epigenetic modifications in the HCT116, SW480, and NCM460 cell lines, DCBLD2-SE was partitioned into four discrete H3K27ac marked sub-enhancer units, designated SE1 to SE4 (Fig. 2j). These four regions displayed robust signals for H3K27ac, P300, DNase I accessibility and ATAC-seq chromatin openness in CRC cells; such features were largely absent in NCM460 control cells. ChIP-qPCR confirmed enrichment of H3K27ac on enhancer clusters ranging from SE1 to SE4 in both HCT116 and SW480 cells (Fig. 3a). Collectively, these experimental data demonstrate that DCBLD2-SE comprises multiple H3K27ac positive sub-enhancer modules and governs DCBLD2 expression in a BRD4-dependent manner.

The constituent active regions of DCBLD2-SE were individually dissected to precisely map its core transcriptional regulatory domain. The results of the luciferase reporter assay revealed a 40-fold increase in the luciferase activity of SE3 in both HCT116 and SW480 cells. Conversely, luciferase activity was not detected in NCM460 cells (Fig. 3b). JQ1 treatment markedly diminished the transcriptional activity driven by the SE3 fragment (Fig. 3c). These findings suggest that SE3 component enhancer has substantially higher enhancer activity than the other three enhancers in HCT116 and SW480 cells. Based on the TFs combination predictions from the hTFtarget database, SE3 is further subdivided into four subregions: SE3-1 to SE3-4 (Fig. S1d). The results of the luciferase experiments revealed that the luciferase activity of SE3-2 increased 38- and 25-fold in HCT116 and SW480 cells, respectively (Fig. 3d). This finding indicates that SE3-2 also has greater enhancer activity. To narrow down the minimal functional fragment, SE3-2 was further divided into regions SE3-2–1 to SE3-2–3 by analyzing their TFs binding sites. Although SE3-2–3 contained almost all of the predicted TFs binding sites, its luciferase activity was not altered (Fig. 3e). No smaller fragment recapitulated the full enhancer potency of SE3-2. Thus, SE3-2 represents the minimal functional unit of DCBLD2-SE, and subsequent studies focused mainly on SE3-2.

### FOSL2 and JUND serve as core TFs governing SE3-2 enhancer activity

**Fig. 4:**
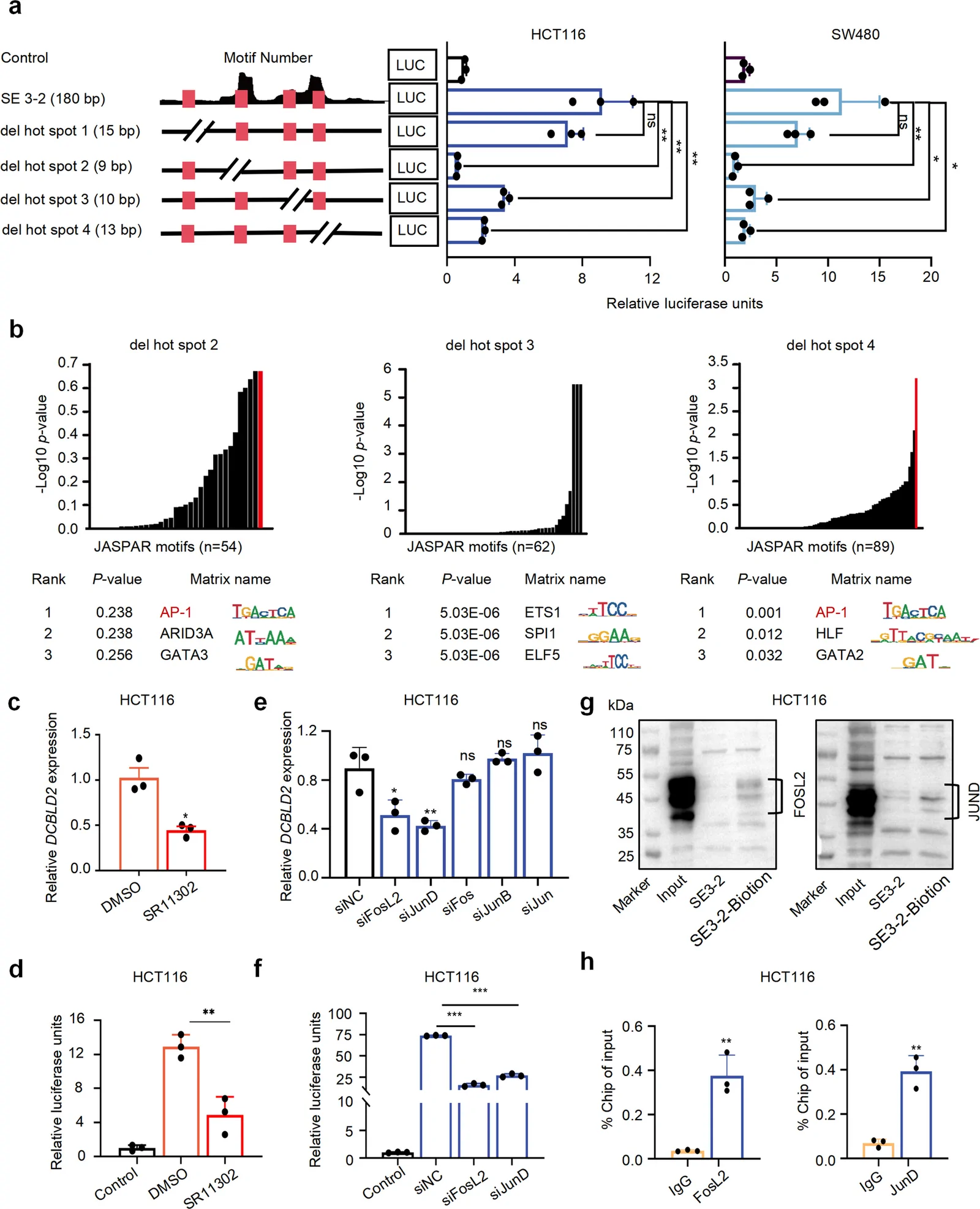
AP-1 binding hot spot motifs within SE3-2 mediate FOSL2/JUND-dependent transcriptional activation of DCBLD2.** a** Dual-luciferase activity was measured after deleting the four predicted TFs binding hotspots (del hot spot 1–4) in SE3-2 (*n* = 3). **b** Volcano plot illustrating TFs binding enrichment predicted from the SE3-2 sequence using the JASPAR database (https://jaspar.elixir.no/). From the hot spot regions, the top three TFs ranked by confidence score (del hot spot 2, *n* = 54; del hot spot 3, *n* = 62; del hot spot 4, *n* = 89). **c** The mRNA expression level of *DCBLD2* was measured using RT-qPCR following treatment with the 1 μM AP-1 inhibitor SR11302. The expression of *GAPDH* was used to standardize the RT-qPCR results; DMSO, as a vehicle control (*n* = 3). **d** Dual luciferase activity of SE3-2 following treatment with 1 μM SR11302 (*n* = 3). **e** After siRNA-mediated knockdown of individual members of the AP-1 family of TFs (siFOS, siJUNB, siJUND, siFOSL2 and siJUN), *DCBLD2* mRNA expression was quantified by RT-qPCR (*n* = 3). The expression of *GAPDH* was used to standardize the RT-qPCR results; siNC, non-targeting negative-control siRNA. **f** Dual-luciferase reporter assay showing SE3-2 activity in HCT116 cells following co-transfection of the SE3-2 construct with siFOSL2, siJUND and siNC (*n* = 3). **g** The biotin-labeled DNA pull-down Western blot experiment was performed to verify the binding of endogenous FOSL2 and JUND to the SE3-2 probe. The input serves as a positive control for nuclear proteins; SE3-2 refers to the unbiotinylated SE3-2 probe, while SE3-2-biotin denotes the biotinylated SE3-2 probe along with nuclear proteins. **h** Endogenous ChIP-qPCR confirming FOSL2 and JUND occupancy at the SE3-2 locus in HCT116 cells. IgG as immunoprecipitation negative control. **a**, **d**, and **f** PGL4.10 empty vector as a control. Three biological replicates’ means are displayed. SEMs are indicated by error bars. With a two-tailed Student’s t test, *P < 0.05, **P < 0.01, ***P < 0.001, and ns, not significant

Motif-prediction analyses based on the JASPAR database were performed to uncover functional DNA-binding motifs residing in the SE3-2 fragment and retrieve candidate interacting TFs. The analyses revealed that four sites within SE3-2 exhibited TFs binding enrichment, and these sites were designated hot spot motifs 1–4 (Fig. S2a, b). Luciferase activity assays showed that reduced reporter activity by approximately 90% following the deletion of hot spot del 2 (Fig. 4a). Ranking candidate transcription factors according to their binding confidence scores across hot spot 2, hot spot 3, and hot spot 4 revealed prominent enrichment of AP-1 family TFs at the hot spot 2 and hot spot 4 loci (Fig. 4b).

To validate the function of AP-1 TFs, the AP-1 inhibitor SR11302 was used to treat CRC cells. The findings indicated that the inhibitor SR11302 decreased *DCBLD2* mRNA expression by 65% and SE3-2 enhancer activity by 62% (Fig. 4c, d). Based on the JASPAR motif score > 0.9 for the AP-1 TFs, JunB, JunD, Fos, FosL1, and FosL2 were selected for siRNA-mediated loss-of-function analysis (Table S4). The results indicated that inhibition of FosL2 and JunD reduced *DCBLD2* mRNA expression by 40% and 50%, respectively, whereas siRNAs targeting other AP-1 subunits produced no notable effects (Fig. 4e and Fig. S2c). Luciferase reporter assays demonstrated that FOSL2 or JUND silencing reduced SE3-2 enhancer activity by 80% and 63% respectively (Fig. 4f). To elucidate the interaction between FOSL2/JUND and SE3-2, DNA pull-down assay was conducted using a biotin-labeled SE3-2 DNA probe. Silver-staining and immunoblot results confirmed that both FOSL2 and JUND associate with SE3-2 (Fig. 4g and Fig. S2d). The ChIP-qPCR results validated that, compared with the IgG negative control, FOSL2 and JUND demonstrated markedly enriched occupancy to the SE3-2 region (Fig. 4h). These findings suggest that AP-1 binding motifs constitute the core functional domain of SE3-2; FOSL2 and JUND occupy this enhancer element to drive *DCBLD2* transcription.

### CRISPR-mediated ablation of DCBLD2-SE reveals its regulatory function in CRC cells

**Fig. 5:**
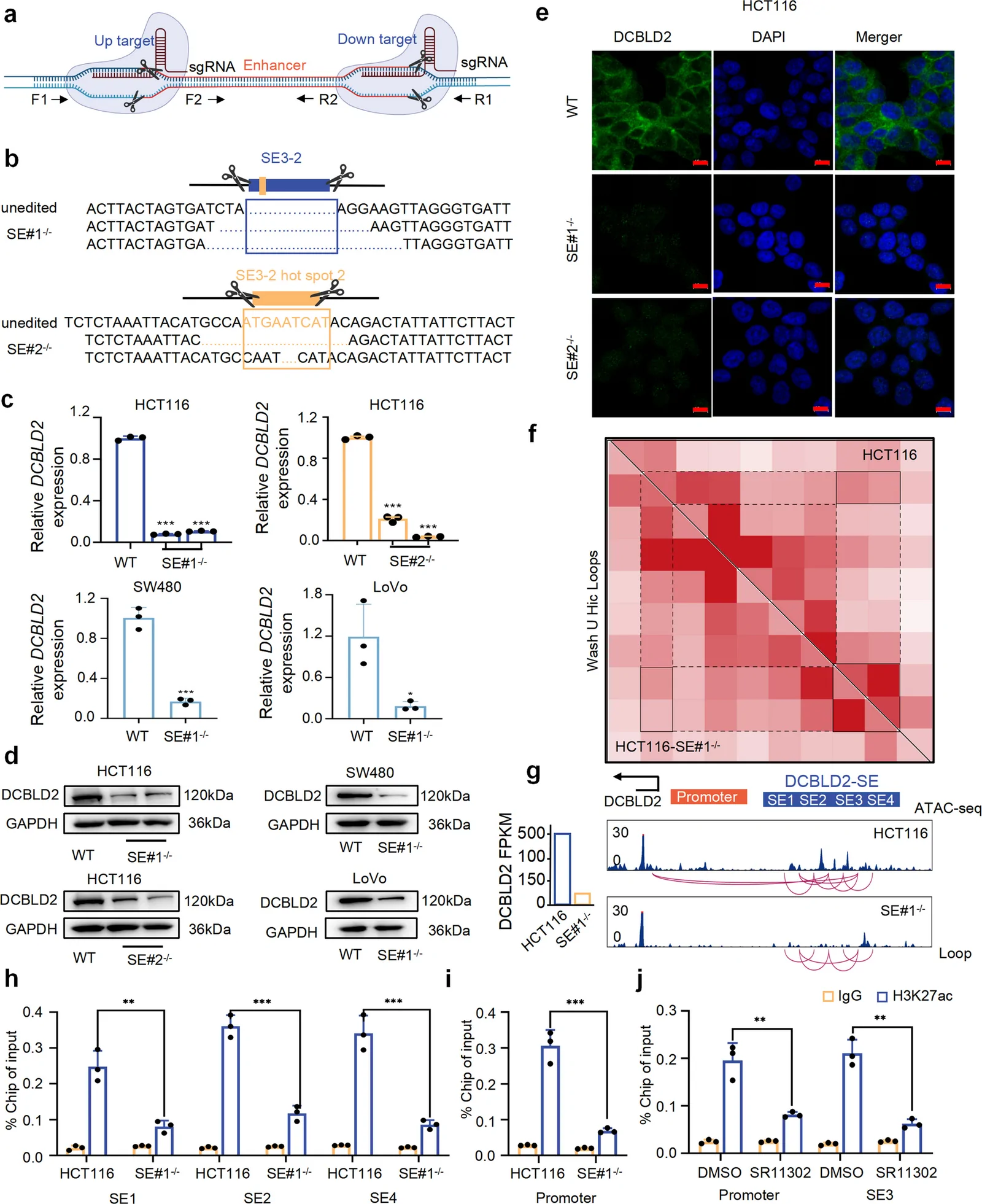
CRISPR/Cas9 deletion of SE3-2 impairs DCBLD2 expression and disrupts local chromatin remodeling.** a** Schematic diagram showing the design of CRISPR/Cas9 sgRNAs for site-specific deletion of the entire SE3-2 (SE#1^−/−^) and AP-1 binding hot spot 2 (SE#2^−/−^) at the DCBLD2-SE locus. The upstream and downstream sgRNA pairs (F1/F2, R1/R2) used for site-specific cleavage are labeled. **b** Generation of HCT116-derived cell lines with genomic deletion of component SE3-2 (SE#1^−/−^) and motif hot spot 2 (SE#2^−/−^) of DCBLD2-SE were deleted. **c** RT-qPCR analysis were performed to determine the relative expression levels of DCBLD2 mRNA in the knockout cell lines HCT116, SW480, and LoVo (*n* = 3). WT, wild type group; SE#1^−/−^, SE3-2 activity-deficient group; SE#2^−/−^, motif hot spot 2 activity-deficient group. *GAPDH* was used as an used for normalization. **d** The expression levels of the DCBLD2 protein in the knockout cell lines HCT116, SW480, and LoVo were determined using Western blot analysis. **e** Immunofluorescence staining of DCBLD2 (green membrane signal) and DAPI nuclear counterstain (blue) in WT, SE#1^−/−^ and SE#2^−/−^ HCT116 cells; Red scale bar, 10 μm. **f** Hi-C chromatin interaction heatmap comparing TAD loop frequency at DCBLD2 locus between WT and SE#1^−/−^ in HCT116. Increased red intensity indicates higher chromatin-looping frequency. **g** ATAC-seq analysis of chromosomal changes in the promoter and enhancer regions of DCBLD2-SE active deletion in HCT116. **h** ChIP-qPCR analysis of H3K27ac histone modifications on residual DCBLD2-SE fragments (SE1, SE2, SE4) in SE#1^−/−^ cells (*n* = 3). **i** ChIP-qPCR analysis of histone marks modification on the DCBLD2 promoter after DCBLD2-SE deletion (*n* = 3). **j** ChIP-qPCR analysis was conducted to assess the modification of histone marks on the SE3 and DCBLD2 promoters following treatment with 1 μM SR11302 (*n* = 3). DMSO was used as the vehicle control. **h-j** IgG as immunoprecipitation negative control. Three biological replicates’ means are displayed. SEMs are indicated by error bars. With a two-tailed Student’s t test, **P < 0.01, ***P < 0.001

The functional role of DCBLD2-SE in CRC cells was validated by generating homozygous knockout cell lines lacking SE3-2 or hot spot del 2 (Fig. 5a, b). In the DCBLD2-SE knockout groups of HCT116, SW480, and LoVo cells, the mRNA expression levels of *DCBLD2* decreased significantly by 90% (Fig. 5c). Western blot analysis revealed that DCBLD2 protein levels were concurrently suppressed (Fig. 5d and Fig. S3a). Immunofluorescence showed that in cell lines lacking DCBLD2-SE, membrane-associated DCBLD2 fluorescent signals were substantially diminished (Fig. 5e and Fig. S3b). Collectively, these findings establish that DCBLD2-SE is essential for sustaining high DCBLD2 transcript and protein abundance in HCT116, SW480 and LoVo cells.

To characterize chromatin landscape changes following DCBLD2-SE ablation, we integrated multi-omics datasets from wild type (WT) and SE3-2 deleted cells. The Hi-C results showed that cells lacking SE activity exhibit a decrease in the frequency of interactions between their enhancer region and the DCBLD2 promoter region. In addition, chromatin interactions within SE were also significantly reduced (Fig. 5f). Furthermore, among the significantly downregulated differentially expressed genes identified in the A/B compartment, loop, and TAD territories, only the *DCBLD2* gene was found to be located on chr 3 (Fig. S3c). KEGG enrichment of differentially expressed genes (DEGs) highlighted perturbation of the PI3K-AKT signaling pathway (Fig. S4a). ATAC-seq motif analysis indicated global attenuation of AP-1 family TFs activity upon DCBLD2-SE ablation (Fig. S4b). ATAC-seq data also showed that in SE activity-deficient cells, the chromatin activity of the component enhancers SE1 and SE2 significantly decreased, while the activating modifications at SE4 remained largely unchanged (Fig. 5g). Upon deletion of SE3, functional cooperation among sub-enhancer modules, indicating functional cooperation among these sub-enhancers. ChIP-qPCR detected SE1, SE2, and SE4 enhancer regions in SE activity-deficient cells, and the H3K27ac enrichment of H3K27ac was 75%, 70%, and 67% lower than that in HCT116 cells, respectively (Fig. 5h). Moreover, the DCBLD2 promoter region exhibited a 77% reduction in H3K27ac modification (Fig. 5i). Inhibition of AP-1 with SR11302 reduced the H3K27ac signal intensity at the SE3-2 site by approximately 70% and at the DCBLD2 promoter site by approximately 60% (Fig. 5j). These results demonstrate that AP-1 inactivation drives widespread chromatin remodeling, thereby modulating the epigenetic activity of both the SE3-2 enhancer and the DCBLD2 promoter.

### DCBLD2-SE drives CRC progression via the CD146/AKT/TNFRSF6B signaling axis

**Fig. 6:**
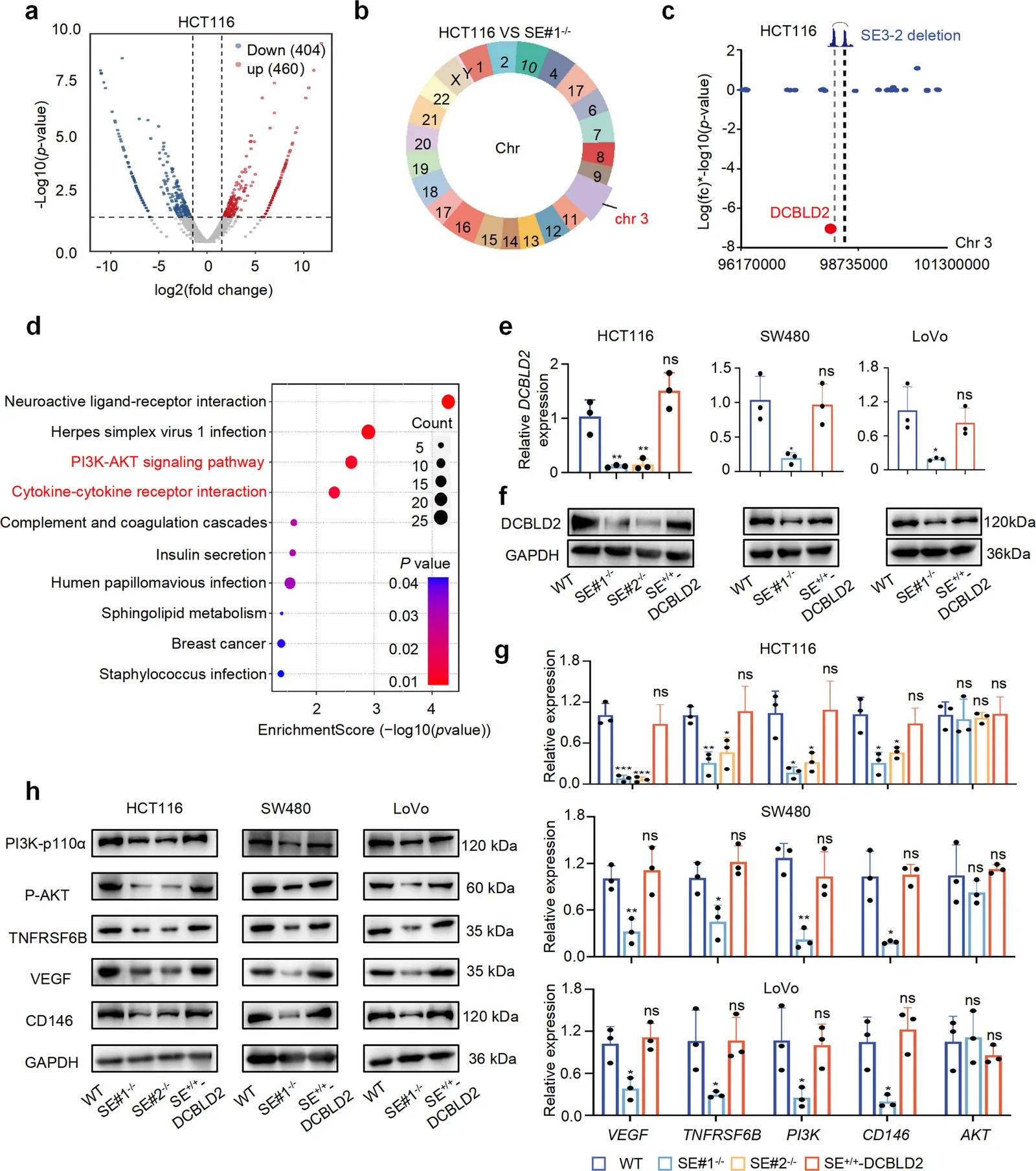
RNA-seq analysis evaluating transcriptomic changes upon DCBLD2-SE deletion in CRC cells.** a** RNA-seq volcano plot of DEGs between HCT116 and SE#1^−/−^ knockout cells. Red dots, significantly upregulated; blue dots, significantly downregulated; black dots, without statistical significance. **b** Chromosomal distribution map of all DEGs identified upon SE3-2 deletion. **c** Heatmap of DEGs residing within the TAD region adjacent to the DCBLD2-SE locus. **d** KEGG pathway enrichment bubble plot for DEGs from the SE3-2 knockout; bubble size corresponds to the number of enriched genes, and the color gradient represents -log₁₀(*P*-value). **e** RT-qPCR analysis of DCBLD2 mRNA levels in SE3-2 knockout cells with exogenous DCBLD2 overexpression (SE⁺/⁺‑DCBLD2) derived from HCT116, SW480, and LoVo cells (*n* = 3). *GAPDH* was used for normalization. **f** Protein expression in SE^+/+^-DCBLD2 cell lines overexpressing DCBLD2 in HCT116, SW480, and LoVo. **g** The mRNA expression levels of genes related to the PI3K-AKT signaling pathway were detected using RT-qPCR (*n* = 3). The expression of *GAPDH* was used to standardize the RT-qPCR results. **h** Western blot analysis of protein abundance of genes related to the PI3K-AKT signaling pathway. Three biological replicates’ means are displayed. SEMs are indicated by error bars. With a two-tailed Student’s t test, *P < 0.05, **P < 0.01, ***P < 0.001, and ns, not significant

RNA-seq profiling was performed to compare gene expression between WT HCT116 and SE#1^−/−^ cells. Based on thresholds of an absolute fold change ≥ 1.5 and a *p*-value < 0.05, a total of 864 DEGs were identified (Table S5). Among them, 460 genes were upregulated, and 404 genes were downregulated (Fig. 6a). These DEGs were distributed across all chrs (Fig. 6b). The upstream and downstream 5 Mb genomic window flanking DCBLD2-SE on chr 3 was further analysed for changes in gene expression. Within this local TAD neighbourhood, only *DCBLD2* exhibited substantial downregulation following SE deletion (Fig. 6c). GO enrichment analysis uncovered perturbed biological processes in signal transduction, protein turnover and cytoskeleton organisation (Fig. S4c). KEGG pathway enrichment analysis revealed that cytokine-cytokine receptor interactions and the associated PI3K-AKT signaling pathway were notable changes (Fig. 6d). Among these pathway members, *TNFRSF6B* was markedly downregulated in the cytokine-cytokine receptor interaction pathway and acts as a downstream effector of PI3K-AKT signaling (Table S5).

To dissect the functional interplay among DCBLD2-SE, DCBLD2, and the PI3K-AKT signaling pathway, *DCBLD2* overexpression cell lines (Fig. 6e, f and Fig. S4d) and DCBLD2 siRNA-knockdown cell models (Fig. S5a, b) were established. RT-qPCR and western blot assays were performed for target-gene validation. Depletion of either *DCBLD2* or DCBLD2-SE suppressed PI3K-AKT signaling in HCT116, SW480, and LoVo cells. The mRNA levels of *PI3K* and *TNFRSF6B* decreased by approximately 60%. *CD146*, as a key adapter between DCBLD2 and PI3K-AKT, showed a significant decrease in mRNA levels. The level of *VEGF* decreased by about 80%, which is consistent with the role of DCBLD2 in angiogenesis (Fig. 6g and Fig. S5c). Protein abundance of these pathway components was concurrently diminished (Fig. 6h and Fig. S5d, e). Ectopic re-expression of *DCBLD2* fully restored the expression of these downstream signaling mediators. Notably, ectopic re-expression of *DCBLD2* fully restored the expression of these downstream signaling mediators. Mechanistically, loss of DCBLD2-SE disrupts DCBLD2-CD146 interaction, blunts PI3K-AKT activity, and reduces TNFRSF6B abundance, a cascade that ultimately compromises CRC cell survival.

### DCBLD2-SE drives malignant phenotypes and in vivo tumor growth of CRC cells by sustaining *DCBLD2* expression

**Fig. 7:**
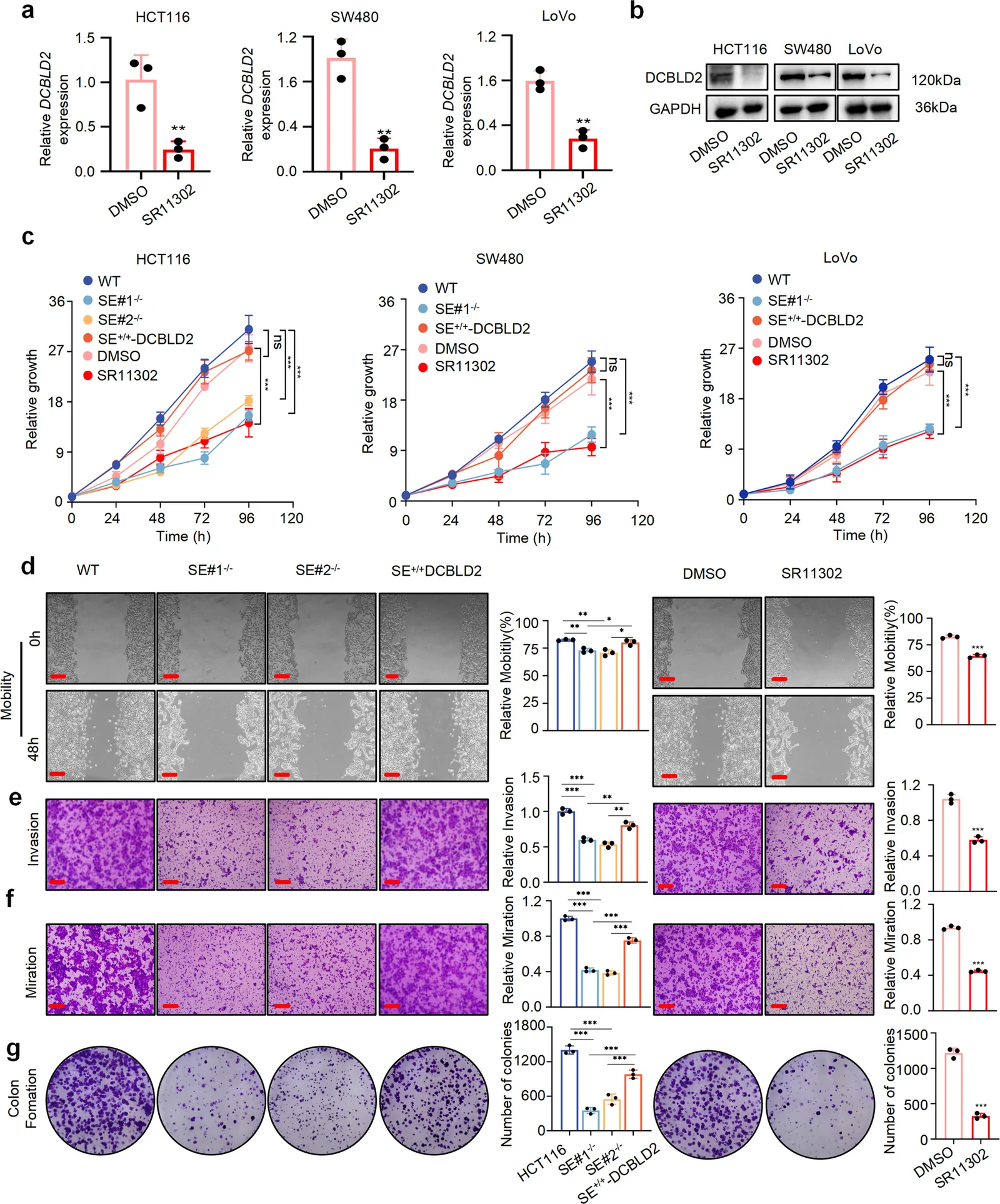
DCBLD2-E drives malignant phenotypes in CRC cells by maintaining DCBLD2 expression.** a** RT-qPCR analysis of DCBLD2 mRNA expression levels in HCT116, SW480, and LoVo cells treated with 1 μM SR11302 (*n* = 3). The expression of GAPDH was used to standardize the RT-qPCR results; DMSO served as the vehicle control. **b** Western blot analysis of DCBLD2 protein expression levels in HCT116, SW480, and LoVo cells following treatment with 1 μM SR11302. **c** Cell proliferation was measured via CCK-8 assays (*n* = 3). Three biological replicates’ means are displayed. SEMs are indicated by error bars. ***P < 0.001 as determined by two-way ANOVA. **d** Wound healing assays were conducted on HCT116 cells at 0 and 48 h to assess their migratory capacity, and the relative migratory capacity was quantified (red scale bar, 100 μm; *n* = 3). **e**, **f** The Transwell assay was used to assess the invasive and migratory capabilities of HCT116 cells. Quantitative analyses of migration and invasion are shown in the figure on the right (scale bar, 100 μm; *n* = 3). **g** The colony formation assay was conducted to evaluate the long-term proliferation capacity of HCT116, and the number of colonies was counted and statistically analyzed (*n* = 3). **a**, **d-g** Three biological replicates’ means are displayed. SEMs are indicated by error bars. With a two-tailed Student’s t test, *P < 0.05, **P < 0.01, ***P < 0.001

To elucidate how DCBLD2-SE loss impacts the malignant behaviour of CRC cells, we first validated that SR11302 suppresses DCBLD2 expression, as confirmed by immunoblotting and RT-qPCR (Fig. 7a, b and Fig. S5f). As shown in Fig. 7c, the deletion of DCBLD2-SE or SR11302 treatment markedly suppressed the proliferation of HCT116, SW480, and LoVo cells after 72 h and 96 h of incubation. Wound healing assays revealed that compared with control cells, the migration rate of DCBLD2-SE-deficient cells was lower, whereas overexpressing DCBLD2 in these cells restored their migration capacity. The effect of the addition of SR11302 recapitulated the migratory defects observed upon DCBLD2-SE deletion (Fig. 7d and Fig. S6a). Transwell migration and invasion assays further showed that DCBLD2-SE ablation or AP-1 inhibition significantly repressed migration and invasion; these defects were rescued by exogenous *DCBLD2* overexpression (Fig. 7e, f and Fig. S6b, c). Moreover, the effect of DCBLD2-SE on cell proliferation was verified by a colony formation assay. The results showed that the colony-forming efficiency of DCBLD2-SE-deficient cells was significantly reduced, whereas DCBLD2 overexpression restored it. SR11302 similarly reduced the colony-forming capacity of HCT116, SW480 and LoVo (Fig. 7g and Fig. S6d). Collectively, these findings ndicate that DCBLD2-SE maintains high DCBLD2 abundance to support in vitro proliferation, migration and invasive properties of HCT116, SW480 and LoVo cells.

**Fig. 8:**
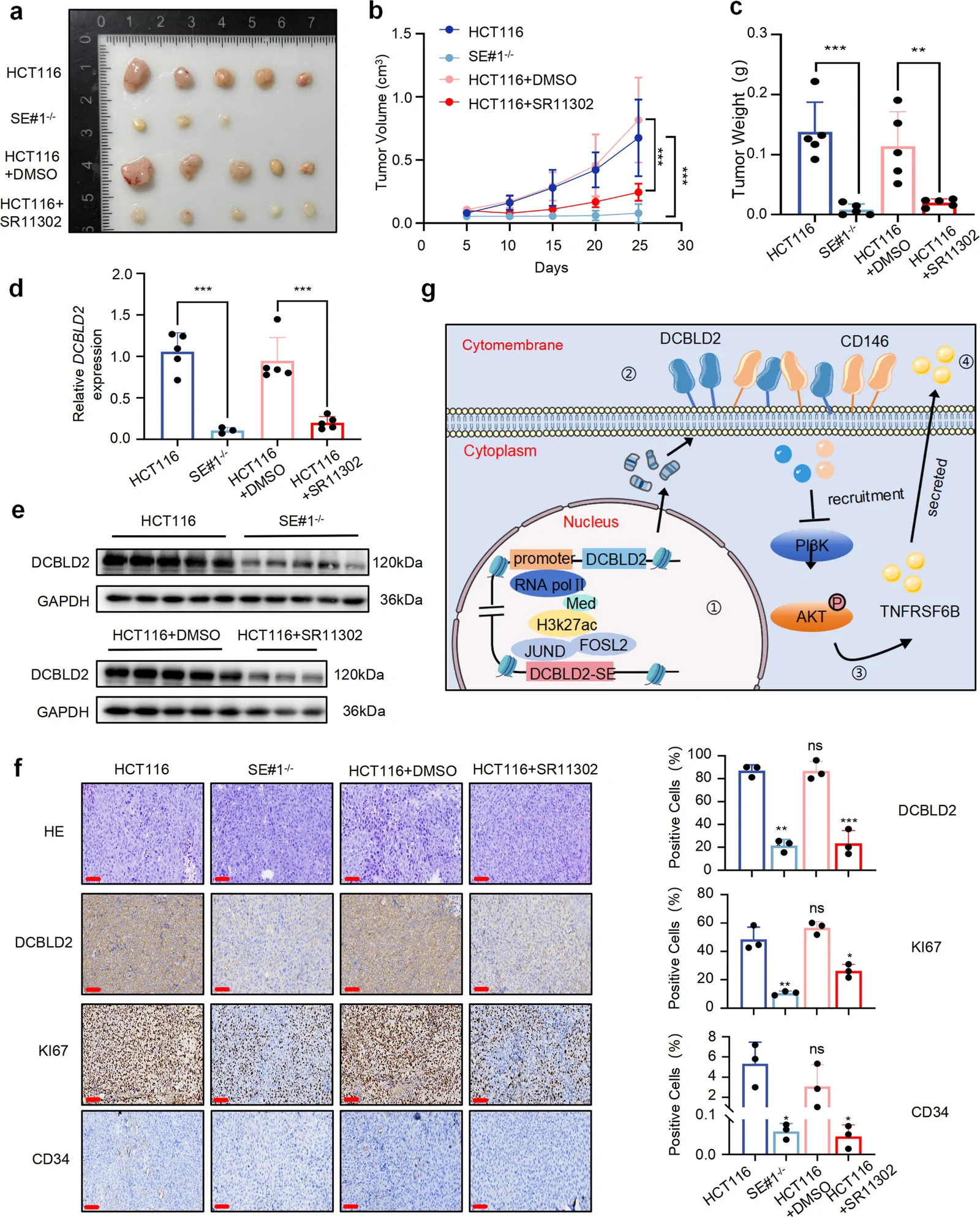
DCBLD2-SE deletion or pharmacological AP-1 inhibition restrains xenograft tumor growth in nude mice in vivo.** a** Representative subcutaneous xenograft tumor photographs from four experimental groups: HCT116, SE#1^−/−^ knockout, DMSO vehicle control (HCT116 + DMSO) and 0.5 mg/kg/day SR11302 AP-1 inhibitor treated (HCT116 + SR11302) nude mice (*n* = 5). **b** Tumor volume growth curve measured every 5 days after subcutaneous implantation (*n* = 5). Five biological replicates’ means are displayed. SEMs are indicated by error bars. ***P < 0.001 as determined by two-way ANOVA. **c** Endpoint dissected tumor weight quantification for each xenograft group (*n* = 5). **d** Detection of DCBLD2 mRNA expression in tumors by RT-qPCR technique (*n* = 5). The expression of *GAPDH* was used to standardize the RT-qPCR results. **e** Western blot detection of DCBLD2 protein expression in tumors. GAPDH as an internal control.** f** Histological staining of paraffin-embedded tumor sections. Morphological examination of HE sections; IHC staining for DCBLD2, KI67, and CD34. Red scale bar, 60 μm. ImageJ was used to score the positive cells (*n* = 3). **g** Schematic model of DCBLD2-SE mediated transcriptional regulation in CRC cells. Three biological replicates’ means are displayed. SEMs are indicated by error bars. With a two-tailed Student’s t test, *P < 0.05, **P < 0.01, ***P < 0.001 and ns, not significant. **c** and **d** Five biological replicates’ means are displayed. SEMs are indicated by error bars. With a two-tailed Student’s t test, *P < 0.05, **P < 0.01, ***P < 0.001

Subcutaneous xenograft assays in nude mice were performed to explore the in vivo oncogenic role of DCBLD2-SE. Female nude mice were randomly assigned to four experimental groups: two groups received subcutaneous injection of WT HCT116 or SE#1^−/−^ cells, while the other two groups were implanted with WT HCT116 cells and subsequently treated with either SR11302 or DMSO vehicle control (Figs. 8a and S7a). Relative to control groups, xenografts from the SE#1^−/−^ group exhibited over 70% reductions in tumor volume and weight. Consistently, SR11302 treatment also markedly suppressed tumor growth (Fig. 8b, c). Both DCBLD2 mRNA and protein levels were substantially downregulated in tumor tissues from SE#1^−/−^ and SR11302 treated mice (Figs. 8d, e and S7b). Notably, no obvious visceral organ toxicity was observed in mice receiving 0.5 mg/kg SR11302 treatment (Fig. S7c, d). Immunohistochemical (IHC) staining of xenograft tissues demonstrated that DCBLD2-SE ablation or SR11302 treatment reduced the immunoreactivity of DCBLD2 and the proliferation marker KI67. Moreover, CD34 staining confirmed diminished intratumoral angiogenesis in DCBLD2-SE deficient tumors (Fig. 8f). Collectively, these in vivo results indicate that DCBLD2-SE modulates the tumor microenvironment and facilitates CRC progression in a *DCBLD2* dependent manner.

## Discussion

Epigenetic modifications are heritable alterations in gene expression that occur without altering the underlying nucleotide sequence of the genome [19]. DNA methylation and histone posttranslational modifications are well-studied layers of cancer epigenetics [20]. During epigenetic reprogramming in tumor cells, canonical promoter regulatory circuits are frequently disrupted [21, 22]. Clustered SEs formed through structural remodeling confer substantially stronger transcriptional activity than isolated individual enhancers. These SE clusters recruit high densities of TFs and RNA polymerase II, maintaining the robust overexpression of oncogenes and driving the survival and proliferation of malignant cells [23, 24]. AP-1, first identified in the 1990 s, is a heterodimeric TFs complex predominantly composed of Jun and Fos family proteins [25]. AP-1 governs core cellular processes including proliferation, migration, invasion and epithelial-mesenchymal transition and acts as a pervasive oncogenic driver across multiple malignancies [26, 27]. Elevated AP-1 activity has been detected in a variety of tumors and transformed cancer cell lines, including pancreatic, colon and lung cancers [28–30]. Activated AP-1 usually binds to the promoter or enhancer regions of its target genes, inducing target gene transcription [31]. Multiple small-molecule AP-1 inhibitors have exhibited anti-tumor potency in preclinical models, rendering AP-1 an attractive therapeutic candidate [32, 33]. Collectively, precise identification of SE-dependent oncogenic regulators in CRC not only provides potential molecular biomarkers for early tumor diagnosis but also establishes a mechanistic basis for developing precision therapeutic strategies targeting SE complexes and their downstream oncogenic signaling networks.

This work identifies a highly active chromatin locus, DCBLD2-SE, which upregulates *DCBLD2* expression to initiate the CD146/AKT/TNFRSF6B signaling cascade and accelerate malignant progression of CRC. Multi-omics integration of ChIP-seq and Hi-C datasets enabled comprehensive profiling of the active histone mark H3K27ac across multiple human cell lines. Although DCBLD2 is known to participate in angiogenesis and cancer progression, its upstream epigenetic regulatory mechanisms in CRC remain remain poorly defined. Our data support DCBLD2 as a direct cis-regulatory target of DCBLD2-SE, and elevated DCBLD2 expression correlates with adverse clinical prognosis in CRC patients.. Mechanistically, DCBLD2 activates the PI3K-AKT pathway via interaction with CD146, thereby promoting tumor cell survival and invasive capacity. These findings are consistent with prior findings in lobular breast cancer [34]. Furthermore, the downregulation of the anti-apoptotic effector *TNFRSF6B* indicates that *DCBLD2* plays a critical role in CRC development. This regulatory cascade mirrors oncogenic signaling circuits in diverse cancer models. Receptor tyrosine kinase‑mediated DCBLD2 modification can amplify PI3K‑AKT oncogenic signaling, while TNF receptor superfamily effectors such as TNFRSF6B may recruit E3 ubiquitin ligase complexes to enhance tumor-promoting outputs [35–37]. Our multi-layered pathway analyses bridge chromatin-level epigenetic control and cytoplasmic oncogenic signal transduction and thereby identifying multiple actionable therapeutic targets.

Hi-C and RNA-seq analyses identify *DCBLD2* as the primary cis-regulatory targe of DCBLD2-SE. Dense clustering of AP-1 transcription factors at DCBLD2-SE may effectively sequester cellular AP-1 pools. This sequestration could indirectly modulate the activity of additional AP-1 dependent enhancers. Such trans-acting effects may extend to enhancers either within the same TAD or across distinct chromatin domains. The synergistic activity of the SE1/SE2/SE3/SE4 submodules within the SE also establishes a synergistic chromatin state that drives *DCBLD2* transcription to levels exceeding those achieved by individual enhancers alone. We identified four AP-1 binding hot spot motifs AP-1 binding motifs (hot spot del 1 to 4) on the SE3-2 enhancer. The SE3-2 fragment can enrich AP-1 family TFs JUND and FOSL2, promoting the transition of chromatin from a chromatin decompaction. Furthermore, chromatin remodeling increased activating histone marks and decreased repressive changes in enhancer elements. This resulted in an increase in the activating modification of the cognate *DCBLD2* promoter, triggering robust transcriptional activation of DCBLD2-SE. This observation aligns with established SE models where SE assemblies concentrate multiple TFs to boost target gene transcription [38]. Our preliminary research suggests that DKK1-SE and Epha2-SE also facilitate oncogenic progression by regulating the transcription of target genes through the recruitment of multiple AP-1 family TFs [39, 40].

Several important limitations of the present study should be acknowledged. First, our analysis of multi-tumor-cohort public H3K27ac ChIP-seq data remains preliminary; expanded pan-cancer epigenomic datasets will be required to robustly the tissue-specific activity of DCBLD2-SE. Second, most in vitro and in vivo functional assays have been conducted in HCT116, SW480, and LoVo cells only. CRC displays profound molecular heterogeneity across consensus molecular subtype (CMS) groups, and we cannot fully conclude that DCBLD2-SE uniformly drives oncogenic signaling across all CMS tumor subgroups [41]. Patient-derived organoids and models representing diverse CMS subtypes will be needed in follow-up investigations. Third, although Hi-C and RNA-seq analysis confirmed that DCBLD2 is the main cis-target of DCBLD2-SE, indirect trans-regulatory effects cannot be completely ruled out. Fourth, our siRNA screen revealed FOSL2 and JUND as the primary drivers, but the binding of AP-1 dimers to enhancers is degenerate and context-dependent [42]. In other signaling contexts (such as MAPK signaling overactivation) [43], FOSL1/JUN heterodimers may also occupy the SE3-2 motif. Future ChIP-seq studies in which multiple TFs are targeted will elucidate the mechanisms underlying the assembly of AP-1 complexes at the DCBLD2-SE site. Fifth, our in vivo xenograft experiments relied on constitutive CRISPR-mediated DCBLD2-SE ablation. Pre-existing proliferative defects in SE-deleted cells may partially impair initial tumor engraftment and confound tumor growth phenotypes. The application of inducible sgRNA systems that delete DCBLD2-SE after tumor establishment will better distinguish early engraftment effects from subsequent tumor progression in future animal studies.

In summary, this study identifies a previously uncharacterized epigenetic regulatory axis in CRC. Mechanistically, DCBLD2-SE recruits FOSL2/JUND complexes to activate the *DCBLD2*-CD146/AKT/TNFRSF6B signaling cascade, thereby driving CRC malignant progression (Fig. 8g). These findings establish DCBLD2-SE and its upstream regulators as promising prognostic biomarkers and therapeutic targets, providing novel mechanistic support for epigenetic precision therapy in CRC.

## Materials and methods

### Cell culture

HCT116, SW480, LoVo and NCM460 cells were cultured in DMEM (GIBCO) supplemented with 10% fetal bovine serum (CellMax) and 1% penicillin/streptomycin (Beyotime). Cells were maintained at 37 ℃ with 5% CO_2_ in a humidified incubator. All cell lines were authenticated via short-tandem-repeat (STR) profiling and routinely tested for mycoplasma contamination.

### Chromatin immunoprecipitation

ChIP assays were conducted with the EZ-ChIP Kit (Upstate). Cells at 80% confluence were crosslinked with 1% formaldehyde and quenched with 0.125 M glycine. Cells were lysed in SDS buffer supplemented with protease inhibitors, and chromatin was sonicated to generate DNA fragments ranging from 200 to 1000 bp. Chromatin samples were incubated with H3K27ac (CST, #8173), JUND (CST, #5000), FOSL2 (CST, #19,967), and IgG control (CST, #3900) antibodies overnight at 4 °C. Immunocomplexes were captured with protein A-agarose beads (CST), followed by washing, reverse cross-linking and DNA purification, after which ChIP-qPCR analysis was performed. The primers used are listed in Table S6.

### Luciferase reporter assays

The pGL4.10-TK (Promega) luciferase vector was used to assess enhancer activity. Mutant fragments generated using the KOD-Plus Mutagenesis Kit (TOYOBO) were subcloned into the vector after digestion with the *Kpn* I and *Xho* I (NEB) restriction enzymes. Luciferase and Renilla plasmids were co-transfected into SW480, HCT116 and NCM460 cells via Lipofectamine 3000 (Invitrogen). Dual-luciferase activity was measured 48 h after transfection. Relative luciferase activity was normalized to Renilla luciferase signals. Primers for plasmid construction and site-directed mutagenesis are listed in Table S6.

### Enhancer deletion mediated by CRISPR/Cas9

The sgRNA targeting the SE region were designed via CRISPRscan, CRISPRdirect and CHOPCHOP tools. Annealed sgRNAs oligonucleotides were ligated into the PX458 vector following double digestion with *Bbs I* and *Bsa I* (NEB). Recombinant plasmids were transfected into cells. GFP-positive cells were sorted 48 h post-transfection and cultured to generate monoclonal SE-knockout cell lines. Putative knockout clones were validated by PCR and Sanger sequencing. The sgRNA and primer sequences are provided in Table S6.

### RT-qPCR assay

The RNAiso Plus Kit (TaKaRa) was used to extract total cellular RNA. Genomic DNA contamination was removed during reverse transcription with the PrimeScript RT Kit (TaKaRa). The cDNA was synthesized from each sample using 1 μg of total RNA as a template. RT-qPCR was performed on an ABI7500 instrument with SYBR Ex Taq (TaKaRa). The thermal cycling program consisted of 95 °C for 30 s, followed by 40 cycles of 95 °C for 5 s and 60 °C for 34 s. Relative mRNA expression were calculated by the 2⁻^ΔΔCt method with *GAPDH* serving as the endogenous reference for normalization.

### Western blot analysis

RIPA buffer (APEXBIO) was used to lyse the cells, and total protein concentrations were quantified using the BCA protein-assay kit. Proteins were separated by 10% SDS-PAGE and transferred onto PVDF membranes. After blocking with 5% non-fat skim-milk, membranes were incubated with the specific primary antibody and the HRP-conjugated secondary antibodies. Chemiluminescent signals were acquired with enhanced ECL detection kit (Beyotime) using Mini-PROTEAN Tetra electrophoresis system (Bio-Rad). Densitometric quantification of immunoblot bands was performed with ImageJ software. Antibody dilutions used for Western blot analysis were as follows: DCBLD2 (Sigma, #HPA016909, dilution 1:1000), FOSL2 (CST, #19,967, dilution 1:1000), phosphorylated AKT (CST, #15,116, dilution 1:1000), VEGF (Proteintech, #19,003–1-AP, dilution 1:1000), PI3K-p110α (Abclonal, #A19742, dilution 1:500), TNFRSF6B (Abclonal, #A0649, dilution 1:500), β-Actin (CST, #4970, dilution 1:1000), CD146 (Abclonal, #A27797, dilution 1:1000), GAPDH (Proteintech, #60,004–1-Ig, dilution 1:1000), JUND (CST, #5000, dilution 1:1000). The pCMV-SPORT6.1-DCBLD2 overexpression vector was obtained from MiaoLing.

### DNA pull-down

Nuclear proteins were extracted from cultured cells using the Nuclear and Cytoplasmic Protein Extraction Kit (Beyotime). Nuclear proteins were pre-cleared with streptavidin magnetic beads (MCE), followed by incubation with biotin-labeled enhancer DNA probes overnight at 4 °C. Following magnetic separation and sequential washing, bound proteins were eluted by boiling. Recovered proteins were subjected to silver staining using the Rapid Silver Staining Kit (Beyotime) as well as Western blot analysis.

### Immunofluorescence staining

HCT116, SW480 and LoVo cells grown on glass coverslips were fixed in 4% paraformaldehyde for 15 min and permeabilized with 0.1% Triton X-100 (Beyotime) for 10 min at room temperature. After 1 h of blocking with 5% BSA (Beyotime), samples were incubated overnight at 4 °C with anti-DCBLD2 primary antibody (Sigma, #HPA016909, dilution 1:50). Following PBS washes, Alexa Fluor 488-conjugated secondary antibody was applied for 1 h in the dark (Abcam, #A-11034, dilution 1:500), and nuclei were counter-stained with DAPI (Beyotime). Coverslips were mounted with anti-fade mounting medium, and confocal images were acquired under identical exposure parameters with scale bars were added to all acquired images.

### RNA-seq analysis

Total RNA was extracted from 3 × 10⁶ logarithmically growing HCT116 and SE#1^‑/‑^ cells. RNA electrophoresis and NanoDrop spectrophotometry were used to assessed RNA integrity and purity of the RNA (RIN ≥ 7.0). Library construction and PE150 sequencing were performed on an Illumina NovaSeq platform. DEGs were screened with fold change absolute values > 1.5 and adjusted *P* < 0.05. GO and KEGG enrichment analyses were implemented using the DAVID database.

### ATAC-seq analysis

After 5 × 10^5 growth phase HCT116 and SE#1^−/−^ cells were prepared as single-cell suspensions, nuclei were obtained by adding lysis buffer. ATAC-seq libraries were then constructed on the Illumina NovaSeq PE150 platform for sequencing. Differential chromatin accessibility peaks were taken as log 2 (fold change) absolute values ≥ 0.5 and *p* < 0.05. Genes linked to differential chromatin peaks were annotated using BLAST, followed by GO and KEGG functional enrichment analyses. Motif recognition was performed via the software MEME and DREME. The annotated TFs binding motifs were aligned using Tomtom software and the JASPAR database. Motif matches were filtered with *P* < 0.0001.

### Hi-C analysis

1 × 10^7 HCT116 and SE#1⁻/⁻ cells were fixed with 37% formaldehyde for 10 min, and cross-linking was quenched with 2 M glycine. The samples were used for library preparation using the BioMarker instrument. Chr interactions, A/B compartment switching, TADs and loops were analyzed via bioinformatics. GO functional and KEGG pathway enrichment analyses were performed on the DEGs. Significance testing was conducted via Fisher's exact test (*P* < 0.05).

### siRNA

siRNAs were synthesized by GenePharma (China). Cells were transfected with siRNAs at a final concentration of 40 nM using Lipofectamine 3000 (Invitrogen). Following 48 h incubation, transfection efficiency was verified by RT-qPCR. The sequences of the siRNAs are shown in Table S6.

### Proliferation and colony formation

HCT116 cells were treated with SR11302 (MCE, #HY15870) at a final concentration of 1 μM. OD_450_ absorbance was measured for five consecutive days using the CCK-8 assay. In the colony formation assay, 500 cells were seeded and cultured for 15 days. Colonies were fixed with 4% paraformaldehyde, stained with 0.1% crystal violet for 30 min, and counted for statistical analysis. The SR11302 stock solution was prepared in DMSO. Cells treated with DMSO at the same concentration served as the vehicle control. The final concentration of DMSO in all culture media was kept below 0.1% to exclude potential solvent-mediated cytotoxicity.

### Wound healing, cell invasion and migration

For Transwell migration/invasion assays, 5 × 10^4^ cells per well were seeded into uncoated (migration) or Matrigel-coated (BD Biosciences) (invasion) upper chambers. Samples were treated with 1 μM of SR11302 (MCE, #HY15870). After 48 h of culture, the cells were fixed with 4% paraformaldehyde and stained with 0.1% crystal violet solution. Cell migration and invasion were visualized under a light microscope and subsequently analyzed using ImageJ software. Wound healing assays were performed using 90% confluent cells. The cells were treated with 1 μM SR11302. Images of the wound healing process were captured at 0 h and 24 h, and the migration rate was calculated for subsequent statistical evaluation. The SR11302 stock solution was prepared in DMSO as the solvent. Cells receiving an equivalent concentration of DMSO served as the vehicle control. The final concentration of DMSO in all culture media was kept below 0.1% to rule out solvent toxicity.

### In vivo tumorigenesis assay

Six-week-old female BALB/c nude mice were obtained from Charles River and maintained in a specific pathogen-free (SPF) environment. 5 × 10^6 HCT116 or SE#1^−/−^ cells mixed with 50 μL of Matrigel were subcutaneously injected into the dorsal flanks of nude mice. Five days post-inoculation, HCT116 tumor-bearing mice were randomized into three groups (*n* = 5). SR11302 was dissolved in DMSO to prepare a 75 mg/mL stock solution protected from light. The working solution was freshly prepared using 5% DMSO, 40% PEG300, 5% Tween 80, and 50% sterile normal saline as solvent systems. SR11302 was intraperitoneally injected into mice for 21 consecutive days at a daily dose of 0.5 mg/kg/day. The vehicle control group was injected with an equal volume of solvent. Tumor length, width and height were measured every 5 days, and tumor volume was calculated as (length × width × height)/2.

### Hematoxylin eosin (HE) staining

Paraffin-embedded tumor tissues were sectioned at 4 μm. Sections were dewaxed with xylene and rehydrated through a graded ethanol series. Following HE staining, dehydrated, cleared, and mounted. Pathological morphological features were visualized under a bright-field microscope.

### IHC staining

Paraffin Sects. (4 μm) were dewaxed and rehydrated, followed by antigen retrieval in sodium citrate buffer (pH 6.0) via microwave treatment for 20 min. Endogenous peroxidase activity was quenched with 3% hydrogen peroxide; non-specific binding was blocked with 3% BSA. Sections were incubated with primary antibodies overnight at 4 °C. After incubating the sections with the HRP-conjugated secondary antibody for 1 h, immunoreactive signals were developed using a DAB staining kit (Solarbio) and counterstained with hematoxylin. Random fields were captured under a microscope, and ImageJ software was used to quantitatively analyze the proportion of antigen-positive cells. Antibody dilutions for IHC are as follows: DCBLD2 (Sigma, #HPA016909, dilution 1:100), KI67 (ACROBiosystems, #HGSS239, dilution 1:100), CD34 (CST, #3569, dilution 1:50).

### Motif analysis

The FASTA sequence of the SE3-2 enhancer was imported into the JASPAR database to predict potential TFs binding sites.

### Statistical analysis

All in vitro experiments were performed with at least three independent biological replicates, and in vivo xenograft studies included five mice per group. Densitometric quantification of Western blot bands was conducted using ImageJ software. Quantitative data are expressed as mean ± standard deviation (SD), and statistical analysis was performed using GraphPad Prism 9. Two-tailed Student’s t-test was used for two-group comparisons; two-way ANOVA with Tukey’s post-hoc test for multiple-group comparisons; Pearson correlation for linear analysis; log-rank test for survival analysis. Statistical significance was defined as *P < 0.05, **P < 0.01, ***P < 0.001. Detailed statistical approaches for individual experiments are described in the corresponding fig. legends.

## Supplementary Information

Supplementary Material 1.

## Data Availability

Public ChIP-seq datasets used in this study are summarized in Table S7 and can be visualized on the UCSC Genome Browser. Raw RNA-seq data generated in this study have been deposited into NCBI Sequence Read Archive (SRA) under accession SUB15402148. Additional numerical experimental datasets supporting the conclusions are available from the corresponding author upon reasonable request.
