# Supplementary material for: The DCBLD2 super-enhancer drives colorectal cancer progression through FOSL2/JUND-mediated activation of the CD146/AKT/TNFRSF6B pathway
Source: Mol Biomed. 2026 Sep 23;7:183. doi: 10.1186/s43556-026-00592-4 (PMC13601434; doi:10.1186/s43556-026-00592-4)
Supplement: Supplementary file 1 [file 43556_2026_592_MOESM1_ESM.docx]

The DCBLD2 super-enhancer drives colorectal cancer progression through FOSL2/JUND-mediated activation of the CD146/AKT/TNFRSF6B pathway

Tong Wu^1^, Lan Shao^2^, Xin Hu^1^, Hongjuan He^1^, Lu Chen^3^, Meiqi Feng^1^, Chengjin Qi^1^, Ziwen Wang^1^, Haoran Yu^4^, Boshu Ji^3^, Yijia Cao^1^, Yan Zhang^1*^, Qiong Wu^1*^

1 School of Life Science and Technology, Faculty of Life Sciences and Medicine, Harbin Institute of Technology, Harbin, China

2 Department of Medicine, Dalian University of Technology, Dalian, China.

3 Department of Pathology, the Second Afﬁliated Hospital of Harbin Medical University, Harbin, China.

4 Department of Biomedical Engineering, School of Medical Information and Engineering, Guangdong Pharmaceutical University, Guangzhou, China.

***Correspondence:** Qiong Wu (kigo@hit.edu.cn) & Yan Zhang (zhangtyo@hit.edu.cn), School of Life Science and Technology, Faculty of Life Sciences and Medicine, Harbin Institute of Technology, Harbin, 150001, China.

**Supplementary Table and Fig.**

**Supplementany Table S1 to S7**

**Table S1** 227 SEs of HCT116 cells included in the SEA database

| SEID | Loci | Name | Length |
| --- | --- | --- | --- |
| 717547 | chr8:125109413-125312260 | hg38_HCT-116_chr8_125109413 | 202847 |
| 717548 | chr3:111869059-111975518 | hg38_HCT-116_chr3_111869059 | 106459 |
| 717549 | chr4:31001437-31115212 | hg38_HCT-116_chr4_31001437 | 113775 |
| 717550 | chr12:88914396-88995392 | hg38_HCT-116_chr12_88914396 | 80996 |
| 717551 | chr8:125635681-125704572 | hg38_HCT-116_chr8_125635681 | 68891 |
| 717552 | chr6:129616795-129776129 | hg38_HCT-116_chr6_129616795 | 159334 |
| 717553 | chr12:65415682-65547436 | hg38_HCT-116_chr12_65415682 | 131754 |
| 717554 | chr4:30886947-30987175 | hg38_HCT-116_chr4_30886947 | 100228 |
| 717555 | chr4:74607986-74684459 | hg38_HCT-116_chr4_74607986 | 76473 |
| 717556 | chr10:112470901-112566159 | hg38_HCT-116_chr10_112470901 | 95258 |
| 717557 | chr4:101086120-101234635 | hg38_HCT-116_chr4_101086120 | 148515 |
| 717558 | chr9:21663246-21730517 | hg38_HCT-116_chr9_21663246 | 67271 |
| 717559 | chr10:28153683-28261933 | hg38_HCT-116_chr10_28153683 | 108250 |
| 717560 | chr2:181763624-181822251 | hg38_HCT-116_chr2_181763624 | 58627 |
| 717561 | chr7:130969925-131045408 | hg38_HCT-116_chr7_130969925 | 75483 |
| 717562 | chr6:151011373-151097869 | hg38_HCT-116_chr6_151011373 | 86496 |
| 717563 | chr14:68074304-68212918 | hg38_HCT-116_chr14_68074304 | 138614 |
| 717564 | chr6:158677664-158746443 | hg38_HCT-116_chr6_158677664 | 68779 |
| 717565 | chr7:129823440-129939611 | hg38_HCT-116_chr7_129823440 | 116171 |
| 717566 | chr1:172825589-172936818 | hg38_HCT-116_chr1_172825589 | 111229 |
| 717567 | chr3:98951067-98987823 | hg38_HCT-116_chr3_98951067 | 36756 |
| 717568 | chr4:11584932-11655392 | hg38_HCT-116_chr4_11584932 | 70460 |
| 717569 | chr3:139287664-139339906 | hg38_HCT-116_chr3_139287664 | 52242 |
| 717570 | chr22:37302904-37347541 | hg38_HCT-116_chr22_37302904 | 44637 |
| 717571 | chr13:97231779-97310653 | hg38_HCT-116_chr13_97231779 | 78874 |
| 717572 | chr2:219681640-219738442 | hg38_HCT-116_chr2_219681640 | 56802 |
| 717573 | chr12:46414293-46497490 | hg38_HCT-116_chr12_46414293 | 83197 |
| 717574 | chr17:59745970-59804107 | hg38_HCT-116_chr17_59745970 | 58137 |
| 717575 | chr1:8103883-8177503 | hg38_HCT-116_chr1_8103883 | 73620 |
| 717576 | chr1:66252978-66318739 | hg38_HCT-116_chr1_66252978 | 65761 |
| 717577 | chr2:223520753-223581545 | hg38_HCT-116_chr2_223520753 | 60792 |
| 717578 | chr2:226019611-226092915 | hg38_HCT-116_chr2_226019611 | 73304 |
| 717579 | chr2:168151836-168231257 | hg38_HCT-116_chr2_168151836 | 79421 |
| 717580 | chr6:56692170-56773944 | hg38_HCT-116_chr6_56692170 | 81774 |
| 717581 | chr4:186801277-186849332 | hg38_HCT-116_chr4_186801277 | 48055 |
| 717582 | chr14:68402193-68602603 | hg38_HCT-116_chr14_68402193 | 200410 |
| 717583 | chr9:21767197-21797934 | hg38_HCT-116_chr9_21767197 | 30737 |
| 717584 | chr7:116414513-116479267 | hg38_HCT-116_chr7_116414513 | 64754 |
| 717585 | chr6:30758782-30823566 | hg38_HCT-116_chr6_30758782 | 64784 |
| 717586 | chr18:3642813-3672951 | hg38_HCT-116_chr18_3642813 | 30138 |
| 717587 | chr3:114635242-114676340 | hg38_HCT-116_chr3_114635242 | 41098 |
| 717588 | chr2:226681023-226785989 | hg38_HCT-116_chr2_226681023 | 104966 |
| 717589 | chr20:62718841-62762536 | hg38_HCT-116_chr20_62718841 | 43695 |
| 717590 | chr4:11487065-11552508 | hg38_HCT-116_chr4_11487065 | 65443 |
| 717591 | chr1:94257565-94331374 | hg38_HCT-116_chr1_94257565 | 73809 |
| 717592 | chr6:129487514-129505493 | hg38_HCT-116_chr6_129487514 | 17979 |
| 717593 | chr7:116723952-116811599 | hg38_HCT-116_chr7_116723952 | 87647 |
| 717594 | chr17:64678063-64735616 | hg38_HCT-116_chr17_64678063 | 57553 |
| 717595 | chr6:144894106-144933753 | hg38_HCT-116_chr6_144894106 | 39647 |
| 717596 | chr20:54096524-54163541 | hg38_HCT-116_chr20_54096524 | 67017 |
| 717597 | chr14:68229364-68308306 | hg38_HCT-116_chr14_68229364 | 78942 |
| 717598 | chr2:223755978-223832502 | hg38_HCT-116_chr2_223755978 | 76524 |
| 717599 | chr9:129547595-129589531 | hg38_HCT-116_chr9_129547595 | 41936 |
| 717600 | chr12:49478612-49521207 | hg38_HCT-116_chr12_49478612 | 42595 |
| 717601 | chr2:231660527-231703406 | hg38_HCT-116_chr2_231660527 | 42879 |
| 717602 | chr1:84337382-84380565 | hg38_HCT-116_chr1_84337382 | 43183 |
| 717603 | chr17:61549250-61584334 | hg38_HCT-116_chr17_61549250 | 35084 |
| 717604 | chr12:13522600-13603300 | hg38_HCT-116_chr12_13522600 | 80700 |
| 717605 | chr5:115911419-115942297 | hg38_HCT-116_chr5_115911419 | 30878 |
| 717606 | chr12:52142398-52176956 | hg38_HCT-116_chr12_52142398 | 34558 |
| 717607 | chr7:80269881-80290062 | hg38_HCT-116_chr7_80269881 | 20181 |
| 717608 | chr8:17834696-17858881 | hg38_HCT-116_chr8_17834696 | 24185 |
| 717609 | chr19:38664625-38736938 | hg38_HCT-116_chr19_38664625 | 72313 |
| 717610 | chr11:108853391-108918094 | hg38_HCT-116_chr11_108853391 | 64703 |
| 717611 | chr2:239243769-239283877 | hg38_HCT-116_chr2_239243769 | 40108 |
| 717612 | chr12:46545771-46560985 | hg38_HCT-116_chr12_46545771 | 15214 |
| 717613 | chr22:24174956-24216066 | hg38_HCT-116_chr22_24174956 | 41110 |
| 717614 | chr5:92549146-92567071 | hg38_HCT-116_chr5_92549146 | 17925 |
| 717615 | chr2:223668175-223707957 | hg38_HCT-116_chr2_223668175 | 39782 |
| 717616 | chr17:15950073-15985364 | hg38_HCT-116_chr17_15950073 | 35291 |
| 717617 | chr17:78311965-78355920 | hg38_HCT-116_chr17_78311965 | 43955 |
| 717618 | chr14:67963589-68019820 | hg38_HCT-116_chr14_67963589 | 56231 |
| 717619 | chr8:127281248-127309828 | hg38_HCT-116_chr8_127281248 | 28580 |
| 717620 | chr10:72288633-72337764 | hg38_HCT-116_chr10_72288633 | 49131 |
| 717621 | chr2:62566930-62581462 | hg38_HCT-116_chr2_62566930 | 14532 |
| 717622 | chrX:45704685-45736873 | hg38_HCT-116_chrX_45704685 | 32188 |
| 717623 | chr5:93144587-93167097 | hg38_HCT-116_chr5_93144587 | 22510 |
| 717624 | chr17:72370073-72432297 | hg38_HCT-116_chr17_72370073 | 62224 |
| 717625 | chr10:90273502-90328118 | hg38_HCT-116_chr10_90273502 | 54616 |
| 717626 | chr15:51840304-51908971 | hg38_HCT-116_chr15_51840304 | 68667 |
| 717627 | chr3:149570837-149610712 | hg38_HCT-116_chr3_149570837 | 39875 |
| 717628 | chr5:142341292-142371046 | hg38_HCT-116_chr5_142341292 | 29754 |
| 717629 | chr3:110565626-110590242 | hg38_HCT-116_chr3_110565626 | 24616 |
| 717630 | chr8:129694228-129727657 | hg38_HCT-116_chr8_129694228 | 33429 |
| 717631 | chr7:130835511-130857902 | hg38_HCT-116_chr7_130835511 | 22391 |
| 717632 | chr4:101008031-101045732 | hg38_HCT-116_chr4_101008031 | 37701 |
| 717633 | chr17:75672980-75709309 | hg38_HCT-116_chr17_75672980 | 36329 |
| 717634 | chr2:231600387-231645253 | hg38_HCT-116_chr2_231600387 | 44866 |
| 717635 | chr17:68376179-68401670 | hg38_HCT-116_chr17_68376179 | 25491 |
| 717636 | chr12:65717063-65732354 | hg38_HCT-116_chr12_65717063 | 15291 |
| 717637 | chr12:78869792-78930164 | hg38_HCT-116_chr12_78869792 | 60372 |
| 717638 | chr12:31469683-31524089 | hg38_HCT-116_chr12_31469683 | 54406 |
| 717639 | chr15:100709714-100734825 | hg38_HCT-116_chr15_100709714 | 25111 |
| 717640 | chr9:107209512-107250479 | hg38_HCT-116_chr9_107209512 | 40967 |
| 717641 | chr8:6572325-6609434 | hg38_HCT-116_chr8_6572325 | 37109 |
| 717642 | chr17:40900589-40923737 | hg38_HCT-116_chr17_40900589 | 23148 |
| 717643 | chr10:110393047-110418814 | hg38_HCT-116_chr10_110393047 | 25767 |
| 717644 | chr12:116491848-116510536 | hg38_HCT-116_chr12_116491848 | 18688 |
| 717645 | chr4:168107481-168168919 | hg38_HCT-116_chr4_168107481 | 61438 |
| 717646 | chr10:99959219-99986939 | hg38_HCT-116_chr10_99959219 | 27720 |
| 717647 | chr6:73565186-73611589 | hg38_HCT-116_chr6_73565186 | 46403 |
| 717648 | chr9:21600473-21620446 | hg38_HCT-116_chr9_21600473 | 19973 |
| 717649 | chr4:186926268-186947956 | hg38_HCT-116_chr4_186926268 | 21688 |
| 717650 | chr1:192966764-192985075 | hg38_HCT-116_chr1_192966764 | 18311 |
| 717651 | chr18:70414258-70435895 | hg38_HCT-116_chr18_70414258 | 21637 |
| 717652 | chr1:27503620-27573301 | hg38_HCT-116_chr1_27503620 | 69681 |
| 717653 | chr4:74530373-74560554 | hg38_HCT-116_chr4_74530373 | 30181 |
| 717654 | chr17:50658365-50701948 | hg38_HCT-116_chr17_50658365 | 43583 |
| 717655 | chr19:13160048-13196603 | hg38_HCT-116_chr19_13160048 | 36555 |
| 717656 | chr17:57859251-57896756 | hg38_HCT-116_chr17_57859251 | 37505 |
| 717657 | chr2:226403170-226442024 | hg38_HCT-116_chr2_226403170 | 38854 |
| 717658 | chr20:35302807-35328538 | hg38_HCT-116_chr20_35302807 | 25731 |
| 717659 | chr15:92798965-92845986 | hg38_HCT-116_chr15_92798965 | 47021 |
| 717660 | chr5:9758-12444 | hg38_HCT-116_chr5_9758 | 2686 |
| 717661 | chr4:11695598-11726119 | hg38_HCT-116_chr4_11695598 | 30521 |
| 717662 | chr7:121273075-121315814 | hg38_HCT-116_chr7_121273075 | 42739 |
| 717663 | chr9:37925683-38049701 | hg38_HCT-116_chr9_37925683 | 124018 |
| 717664 | chr21:31312149-31373554 | hg38_HCT-116_chr21_31312149 | 61405 |
| 717665 | chr22:30194330-30230130 | hg38_HCT-116_chr22_30194330 | 35800 |
| 717666 | chr1:168389340-168411387 | hg38_HCT-116_chr1_168389340 | 22047 |
| 717667 | chr2:226465605-226490891 | hg38_HCT-116_chr2_226465605 | 25286 |
| 717668 | chr7:76280979-76323651 | hg38_HCT-116_chr7_76280979 | 42672 |
| 717669 | chr12:120224141-120242149 | hg38_HCT-116_chr12_120224141 | 18008 |
| 717670 | chr3:152015926-152030650 | hg38_HCT-116_chr3_152015926 | 14724 |
| 717671 | chr16:69015959-69090249 | hg38_HCT-116_chr16_69015959 | 74290 |
| 717672 | chr20:50533811-50574009 | hg38_HCT-116_chr20_50533811 | 40198 |
| 717673 | chr10:124150564-124157535 | hg38_HCT-116_chr10_124150564 | 6971 |
| 717674 | chr5:142815304-142831288 | hg38_HCT-116_chr5_142815304 | 15984 |
| 717675 | chr17:61489486-61507355 | hg38_HCT-116_chr17_61489486 | 17869 |
| 717676 | chr10:73885699-73900653 | hg38_HCT-116_chr10_73885699 | 14954 |
| 717677 | chr12:12802370-12860380 | hg38_HCT-116_chr12_12802370 | 58010 |
| 717678 | chr1:84243449-84273925 | hg38_HCT-116_chr1_84243449 | 30476 |
| 717679 | chr12:15681256-15708751 | hg38_HCT-116_chr12_15681256 | 27495 |
| 717680 | chr12:65299048-65340723 | hg38_HCT-116_chr12_65299048 | 41675 |
| 717681 | chr17:81055983-81122113 | hg38_HCT-116_chr17_81055983 | 66130 |
| 717682 | chr11:35004454-35014272 | hg38_HCT-116_chr11_35004454 | 9818 |
| 717683 | chr12:27606634-27629776 | hg38_HCT-116_chr12_27606634 | 23142 |
| 717684 | chr20:63676318-63693508 | hg38_HCT-116_chr20_63676318 | 17190 |
| 717685 | chr4:142352201-142410025 | hg38_HCT-116_chr4_142352201 | 57824 |
| 717686 | chr4:41140960-41163818 | hg38_HCT-116_chr4_41140960 | 22858 |
| 717687 | chr1:202027968-202087508 | hg38_HCT-116_chr1_202027968 | 59540 |
| 717688 | chr11:57771226-57801745 | hg38_HCT-116_chr11_57771226 | 30519 |
| 717689 | chr7:116338652-116360780 | hg38_HCT-116_chr7_116338652 | 22128 |
| 717690 | chr1:68375144-68397250 | hg38_HCT-116_chr1_68375144 | 22106 |
| 717691 | chr2:84918083-84961228 | hg38_HCT-116_chr2_84918083 | 43145 |
| 717692 | chr10:28048404-28068291 | hg38_HCT-116_chr10_28048404 | 19887 |
| 717693 | chr8:61749447-61769383 | hg38_HCT-116_chr8_61749447 | 19936 |
| 717694 | chr17:17007515-17033414 | hg38_HCT-116_chr17_17007515 | 25899 |
| 717695 | chr5:93076060-93103513 | hg38_HCT-116_chr5_93076060 | 27453 |
| 717696 | chr6:142005785-142017534 | hg38_HCT-116_chr6_142005785 | 11749 |
| 717697 | chr16:69512662-69536091 | hg38_HCT-116_chr16_69512662 | 23429 |
| 717698 | chr13:110943382-110974833 | hg38_HCT-116_chr13_110943382 | 31451 |
| 717699 | chr20:40990197-41013496 | hg38_HCT-116_chr20_40990197 | 23299 |
| 717700 | chr14:68806593-68825919 | hg38_HCT-116_chr14_68806593 | 19326 |
| 717701 | chr1:42922016-42949032 | hg38_HCT-116_chr1_42922016 | 27016 |
| 717702 | chr13:100380517-100398803 | hg38_HCT-116_chr13_100380517 | 18286 |
| 717703 | chr20:49673296-49704198 | hg38_HCT-116_chr20_49673296 | 30902 |
| 717704 | chr6:20996395-21027779 | hg38_HCT-116_chr6_20996395 | 31384 |
| 717705 | chr15:60353299-60383994 | hg38_HCT-116_chr15_60353299 | 30695 |
| 717706 | chr10:70556911-70598297 | hg38_HCT-116_chr10_70556911 | 41386 |
| 717707 | chr10:99740904-99771063 | hg38_HCT-116_chr10_99740904 | 30159 |
| 717708 | chr3:45089635-45130125 | hg38_HCT-116_chr3_45089635 | 40490 |
| 717709 | chr4:168069427-168088385 | hg38_HCT-116_chr4_168069427 | 18958 |
| 717710 | chr11:27925820-27937992 | hg38_HCT-116_chr11_27925820 | 12172 |
| 717711 | chr17:75075173-75081664 | hg38_HCT-116_chr17_75075173 | 6491 |
| 717712 | chr3:114541929-114612191 | hg38_HCT-116_chr3_114541929 | 70262 |
| 717713 | chr8:109588723-109623090 | hg38_HCT-116_chr8_109588723 | 34367 |
| 717714 | chr14:55100881-55110391 | hg38_HCT-116_chr14_55100881 | 9510 |
| 717715 | chr10:119303142-119319485 | hg38_HCT-116_chr10_119303142 | 16343 |
| 717716 | chr10:103887134-103913220 | hg38_HCT-116_chr10_103887134 | 26086 |
| 717717 | chr21:6367082-6375880 | hg38_HCT-116_chr21_6367082 | 8798 |
| 717718 | chr2:64829689-64842593 | hg38_HCT-116_chr2_64829689 | 12904 |
| 717719 | chr2:161120009-161132573 | hg38_HCT-116_chr2_161120009 | 12564 |
| 717720 | chr3:30653643-30693940 | hg38_HCT-116_chr3_30653643 | 40297 |
| 717721 | chr16:89991764-90000685 | hg38_HCT-116_chr16_89991764 | 8921 |
| 717722 | chr8:125543181-125559989 | hg38_HCT-116_chr8_125543181 | 16808 |
| 717723 | chrX:45792982-45844926 | hg38_HCT-116_chrX_45792982 | 51944 |
| 717724 | chr3:177343567-177361605 | hg38_HCT-116_chr3_177343567 | 18038 |
| 717725 | chr14:53838149-53856521 | hg38_HCT-116_chr14_53838149 | 18372 |
| 717726 | chr13:109241886-109275436 | hg38_HCT-116_chr13_109241886 | 33550 |
| 717727 | chr5:93182628-93196580 | hg38_HCT-116_chr5_93182628 | 13952 |
| 717728 | chr18:26476582-26485067 | hg38_HCT-116_chr18_26476582 | 8485 |
| 717729 | chr10:90578189-90594539 | hg38_HCT-116_chr10_90578189 | 16350 |
| 717730 | chr19:18284190-18314625 | hg38_HCT-116_chr19_18284190 | 30435 |
| 717731 | chr17:67417163-67442327 | hg38_HCT-116_chr17_67417163 | 25164 |
| 717732 | chr6:144796058-144821712 | hg38_HCT-116_chr6_144796058 | 25654 |
| 717733 | chr11:102596493-102614122 | hg38_HCT-116_chr11_102596493 | 17629 |
| 717734 | chr9:42420125-42438904 | hg38_HCT-116_chr9_42420125 | 18779 |
| 717735 | chr2:226575291-226589705 | hg38_HCT-116_chr2_226575291 | 14414 |
| 717736 | chr3:151968512-151980447 | hg38_HCT-116_chr3_151968512 | 11935 |
| 717737 | chr19:48483868-48504320 | hg38_HCT-116_chr19_48483868 | 20452 |
| 717738 | chr2:230773006-230797573 | hg38_HCT-116_chr2_230773006 | 24567 |
| 717739 | chr17:72974470-72987192 | hg38_HCT-116_chr17_72974470 | 12722 |
| 717740 | chr20:53619020-53625336 | hg38_HCT-116_chr20_53619020 | 6316 |
| 717741 | chrX:134868284-134910237 | hg38_HCT-116_chrX_134868284 | 41953 |
| 717742 | chr17:48235021-48248477 | hg38_HCT-116_chr17_48235021 | 13456 |
| 717743 | chr10:90218557-90234789 | hg38_HCT-116_chr10_90218557 | 16232 |
| 717744 | chr11:12683764-12699283 | hg38_HCT-116_chr11_12683764 | 15519 |
| 717745 | chr18:77063988-77130142 | hg38_HCT-116_chr18_77063988 | 66154 |
| 717746 | chr4:112165862-112176220 | hg38_HCT-116_chr4_112165862 | 10358 |
| 717747 | chr1:16161213-16190007 | hg38_HCT-116_chr1_16161213 | 28794 |
| 717748 | chr19:47134310-47212153 | hg38_HCT-116_chr19_47134310 | 77843 |
| 717749 | chr11:12720532-12729058 | hg38_HCT-116_chr11_12720532 | 8526 |
| 717750 | chr6:35583488-35608795 | hg38_HCT-116_chr6_35583488 | 25307 |
| 717751 | chr14:54165935-54192190 | hg38_HCT-116_chr14_54165935 | 26255 |
| 717752 | chr4:94471243-94562523 | hg38_HCT-116_chr4_94471243 | 91280 |
| 717753 | chr7:121527533-121558424 | hg38_HCT-116_chr7_121527533 | 30891 |
| 717754 | chr10:119251654-119273752 | hg38_HCT-116_chr10_119251654 | 22098 |
| 717755 | chr3:104425642-104462347 | hg38_HCT-116_chr3_104425642 | 36705 |
| 717756 | chr14:61557679-61567173 | hg38_HCT-116_chr14_61557679 | 9494 |
| 717757 | chr20:17834167-17851925 | hg38_HCT-116_chr20_17834167 | 17758 |
| 717758 | chr10:80464329-80511960 | hg38_HCT-116_chr10_80464329 | 47631 |
| 717759 | chr15:67072402-67117902 | hg38_HCT-116_chr15_67072402 | 45500 |
| 717760 | chr6:158024966-158070658 | hg38_HCT-116_chr6_158024966 | 45692 |
| 717761 | chr15:44153652-44164474 | hg38_HCT-116_chr15_44153652 | 10822 |
| 717762 | chr7:158801341-158823879 | hg38_HCT-116_chr7_158801341 | 22538 |
| 717763 | chr17:920259-939823 | hg38_HCT-116_chr17_920259 | 19564 |
| 717764 | chr10:73955768-73969386 | hg38_HCT-116_chr10_73955768 | 13618 |
| 717765 | chr3:174437325-174443981 | hg38_HCT-116_chr3_174437325 | 6656 |
| 717766 | chrX:23778426-23830584 | hg38_HCT-116_chrX_23778426 | 52158 |
| 717767 | chr8:8287014-8312355 | hg38_HCT-116_chr8_8287014 | 25341 |
| 717768 | chr8:127325916-127350360 | hg38_HCT-116_chr8_127325916 | 24444 |
| 717769 | chr20:51737387-51762520 | hg38_HCT-116_chr20_51737387 | 25133 |
| 717770 | chr4:97490761-97501024 | hg38_HCT-116_chr4_97490761 | 10263 |
| 717771 | chr11:10513466-10533224 | hg38_HCT-116_chr11_10513466 | 19758 |
| 717772 | chr5:76828736-76841609 | hg38_HCT-116_chr5_76828736 | 12873 |
| 717773 | chr10:22814836-22827170 | hg38_HCT-116_chr10_22814836 | 12334 |

**Table S2.** SEs containing the H3K27ac modification and having high-frequency promoter-enhancer interactions

| Sample ID | Species | Data source | Tissue type |
| --- | --- | --- | --- |
| Sample_01_0034 | Human | ENCODE | Colon |
| Sample_01_0114 | Human | ENCODE | Colon |
| Sample_02_0141 | Human | NCBI GEO/SRA | Colon |
| Sample_02_0142 | Human | NCBI GEO/SRA | Colon |
| Sample_02_0143 | Human | NCBI GEO/SRA | Colon |
| Sample_02_0352 | Human | NCBI GEO/SRA | Colon |
| Sample_02_0426 | Human | NCBI GEO/SRA | Colon |
| Sample_02_0427 | Human | NCBI GEO/SRA | Colon |
| Sample_02_0458 | Human | NCBI GEO/SRA | Colon |
| Sample_02_0598 | Human | NCBI GEO/SRA | Colon |
| Sample_02_0599 | Human | NCBI GEO/SRA | Colon |
| Sample_02_0607 | Human | NCBI GEO/SRA | Colon |
| Sample_02_0608 | Human | NCBI GEO/SRA | Colon |
| Sample_02_0870 | Human | NCBI GEO/SRA | Colon |
| Sample_02_0871 | Human | NCBI GEO/SRA | Colon |
| Sample_02_0872 | Human | NCBI GEO/SRA | Colon |
| Sample_02_0873 | Human | NCBI GEO/SRA | Colon |
| Sample_02_0874 | Human | NCBI GEO/SRA | Colon |
| Sample_02_1029 | Human | NCBI GEO/SRA | Colon |
| Sample_02_1247 | Human | NCBI GEO/SRA | Colon |

**Table S3.** Top 15 SEs Target Genes in the Database

| NO. | Target Genei | SE RANK | GENE FPKM |
| --- | --- | --- | --- |
| 1 | ERRFI1 | 2 | 278.1065 |
| 2 | CYB5B | 19 | 148.5458 |
| 3 | SCD | 55 | 314.9441 |
| 4 | ACTN4 | 70 | 150.3695 |
| 5 | ZFP36L1 | 86 | 133.6993 |
| 6 | KRT19 | 91 | 577.7482 |
| 7 | FAT1 | 103 | 260.3941 |
| 8 | PTMA | 107 | 1460.2011 |
| 9 | DCBLD2 | 113 | 167.7400 |
| 10 | SLC38A2 | 162 | 145.2025 |

**Table S4. Predicted enrichment of AP-1 family TFs in the SE3 active site**

| **Name** | **Score** | **Relative score** | **Predicted sequence** |
| --- | --- | --- | --- |
| MA0476.1.FOS | 13.1295 | 0.9640481 | TATGATTCATT |
| MA0491.1.JUND | 11.325999 | 0.9380411 | TATGATTCATT |
| MA0490.1.JUNB | 10.999346 | 0.93210965 | GTATGATTCAT |
| MA0477.1.FOSL1 | 10.181112 | 0.9098574 | AATGAATCATA |
| MA0478.1.FOSL2 | 10.035912 | 0.9119051 | GTATGATTCAT |
| MA0476.1.FOS | 9.8348875 | 0.9201075 | AATGAATCATA |
| MA0490.1.JUNB | 9.474003 | 0.9123851 | GGGTGATTCAA |
| MA0488.1.JUN | 7.3544474 | 0.867818 | GAAGTGAGGCAAG |
| MA0476.1.FOS | 7.1168947 | 0.88385725 | GGTGATTCAAC |
| MA0490.1.JUNB | 7.0761766 | 0.8813784 | TTATGACTAAA |
| MA0476.1.FOS | 7.032565 | 0.88273257 | GTTTAGTCATA |
| MA0478.1.FOSL2 | 7.030086 | 0.87034935 | CAATGAATCAT |
| MA0478.1.FOSL2 | 6.909088 | 0.86867654 | AGATGACACAT |
| MA0490.1.JUNB | 6.4946976 | 0.87385917 | AGATGACACAT |
| MA0490.1.JUNB | 6.446391 | 0.87323445 | CAATGAATCAT |
| MA0477.1.FOSL1 | 6.3432326 | 0.8566979 | AATGTGTCATC |
| MA0476.1.FOS | 6.277389 | 0.87266064 | GTTGAATCACC |
| MA0491.1.JUND | 6.2307787 | 0.8813865 | AATGAATCATA |
| MA0477.1.FOSL1 | 6.2136297 | 0.8549028 | GTTGAATCACC |
| MA0491.1.JUND | 6.1373253 | 0.8803474 | AATGTGTCATC |
| MA0477.1.FOSL1 | 5.7319026 | 0.84823024 | GATGACACATT |
| MA0476.1.FOS | 5.6693215 | 0.8645508 | GATGACACATT |
| MA0491.1.JUND | 5.5478396 | 0.8737927 | GATGACACATT |
| MA0488.1.JUN | 5.3906646 | 0.84643024 | ATAATGGTGACAT |
| MA0478.1.FOSL2 | 5.371586 | 0.8474205 | GGGTGATTCAA |
| MA0478.1.FOSL2 | 5.323965 | 0.8467622 | AAATGTGTCAT |
| MA0477.1.FOSL1 | 5.215407 | 0.84107614 | TATGATTCATT |
| MA0490.1.JUNB | 5.11335 | 0.8559967 | AAATGTGTCAT |
| MA0476.1.FOS | 5.110845 | 0.85710233 | TCTGTCTCATA |
| MA0488.1.JUN | 5.0400457 | 0.8426116 | CTAGTGATCTAAG |
| MA0476.1.FOS | 4.876962 | 0.85398304 | AATGTGTCATC |
| MA0478.1.FOSL2 | 4.3685207 | 0.8335531 | TTATGAGACAG |
| MA0477.1.FOSL1 | 4.3524356 | 0.8291229 | TATGAGACAGA |
| MA0490.1.JUNB | 4.0127835 | 0.8417651 | TTATGAGACAG |
| MA0477.1.FOSL1 | 3.9570951 | 0.82364696 | TATGACTAAAC |
| MA0491.1.JUND | 3.9331596 | 0.85583884 | GGTGATTCAAC |
| MA0477.1.FOSL1 | 3.796508 | 0.82142264 | TCTGTCTCATA |
| MA0491.1.JUND | 3.7744777 | 0.8540744 | TCTGTCTCATA |
| MA0488.1.JUN | 3.5407152 | 0.82628226 | GGAATGATGTATG |
| MA0478.1.FOSL2 | 3.5153558 | 0.821758 | GGGTGATTTAC |
| MA0477.1.FOSL1 | 3.4842124 | 0.8170969 | GGTGATTCAAC |
| MA0478.1.FOSL2 | 3.4420054 | 0.8207439 | GGTTGAATCAC |
| MA0477.1.FOSL1 | 3.0798688 | 0.81149626 | GTTTAGTCATA |
| MA0491.1.JUND | 2.543658 | 0.8403887 | TATGACTAAAC |
| MA0490.1.JUNB | 2.517596 | 0.8224305 | GGGTGATTTAC |
| MA0488.1.JUN | 2.1988463 | 0.8116678 | ACCATTAGGAAAT |
| MA0476.1.FOS | 2.1460674 | 0.81756073 | GGTGATTTACT |
| MA0478.1.FOSL2 | 2.129201 | 0.8025943 | TTATGACTAAA |
| MA0478.1.FOSL2 | 2.0196803 | 0.80108017 | TCTTGCCTCAC |
| MA0490.1.JUNB | 1.8210078 | 0.81342274 | AAGTGAGGCAA |
| MA0488.1.JUN | 1.7005286 | 0.8062406 | TGGGTGATTCAAC |
| MA0491.1.JUND | 0.98316723 | 0.8230374 | TATGAGACAGA |
| MA0490.1.JUNB | 0.8541077 | 0.8009196 | CTCTGTCTCAT |
| MA0491.1.JUND | -0.33515668 | 0.80837864 | GGTGATTTACT |

**Table S5.** DEGs obtained by RNA-seq analysis

| gene_symbol | HCT116 | SE | PValue | log2FC |
| --- | --- | --- | --- | --- |
| AC026954.2 | 8.60E-05 | 0.404961 | 0.000269148 | 5.00037068 |
| DCBLD2 | 167.74009 | 18.918271 | 0.000555105 | -3.111752029 |
| TNFRSF6B | 107.193237 | 27.208414 | 0.022726576 | -1.966761054 |
| NPW | 91.135374 | 47.039068 | 0.04996005 | -1.678355843 |
| CHCHD10 | 58.501482 | 18.615765 | 0.031865184 | -1.848126197 |
| WNT16 | 29.227062 | 4.665348 | 0.002819626 | -2.646576876 |
| RPL36A-HNRNPH2 | 27.650135 | 0.81309 | 7.69E-07 | -5.023602116 |
| CISD2 | 26.78322 | 5.523021 | 0.039708157 | -1.769634219 |
| HOXB8 | 25.773162 | 5.024389 | 0.008586196 | -2.304980895 |
| RBM12 | 20.053671 | 6.160679 | 0.024468065 | -1.940689041 |
| CREB3L2 | 19.790367 | 6.146599 | 0.021281053 | -1.989909894 |
| HAS3 | 18.528004 | 26.239293 | 0.046973043 | 1.70209674 |
| Human_newGene_12645 | 15.437329 | 4.170369 | 0.042114793 | -1.743237328 |
| ZKSCAN1 | 14.574331 | 3.995839 | 0.016484473 | -2.077873933 |
| TMEM250 | 14.369852 | 4.242138 | 0.01379356 | -2.146275204 |
| RGS2 | 13.673764 | 3.068606 | 0.014365142 | -2.142916899 |
| GATA2 | 13.311605 | 2.958593 | 0.006939103 | -2.376165491 |
| SPC24 | 11.948344 | 7.969337 | 0.046063437 | -1.720246697 |
| MATR3 | 11.795015 | 47.416747 | 0.036682008 | 1.793466824 |
| AHNAK2 | 11.27513 | 7.542619 | 0.001851885 | -2.767423161 |
| AC006486.1 | 10.976687 | 0.99861 | 0.00034086 | -3.426363002 |
| MYC | 10.128631 | 42.255417 | 0.004646459 | 2.494662548 |
| SLC6A6 | 9.733776 | 19.482756 | 0.031812495 | 1.846666277 |
| BLOC1S6 | 9.090901 | 29.872115 | 0.002561253 | 2.676452403 |
| ZNF286A | 8.869335 | 5.299104 | 0.039547104 | -1.772451435 |
| BCL2L11 | 8.624093 | 2.933528 | 0.042235369 | -1.746755078 |
| P2RY11 | 8.425525 | 0.697612 | 0.000141287 | -3.56378279 |
| CSTA | 8.366546 | 28.872692 | 0.038207925 | 1.791746004 |
| SERF1A | 8.283185 | 0.817386 | 0.000146629 | -3.608066085 |
| LEPROT | 8.051431 | 3.08144 | 0.034392612 | -1.824660037 |
| ST6GALNAC2 | 7.832062 | 1.911746 | 0.008830553 | -2.311232951 |
| CDRT4 | 7.748159 | 1.827182 | 0.0036831 | -2.609483725 |
| PRRG1 | 7.375142 | 2.154545 | 0.021216847 | -2.002759838 |
| C7orf25 | 7.337167 | 2.251182 | 0.020827787 | -2.031544247 |
| ZNF24 | 7.059077 | 25.824157 | 0.02831441 | 1.887976182 |
| AP003419.1 | 6.865686 | 0.281062 | 1.31E-05 | -4.541338073 |
| TJP2 | 6.016843 | 10.390541 | 0.006169983 | 2.416772655 |
| F8A2 | 5.655727 | 1.524515 | 0.034849077 | -1.867303658 |
| Human_newGene_22686 | 5.533838 | 1.049803 | 0.012758682 | -2.179886442 |
| SAPCD1 | 5.258681 | 0.428749 | 0.000199705 | -3.609664151 |
| QKI | 5.069943 | 5.764818 | 0.038796138 | -1.778916873 |
| AL662899.2 | 5.003654 | 0.347258 | 2.92E-05 | -4.032440695 |
| MAP3K13 | 4.634239 | 12.027172 | 0.038776347 | 1.787317745 |
| ZNF28 | 4.254176 | 2.506096 | 0.000735064 | -3.097026434 |
| ZNF266 | 4.121521 | 0.538398 | 0.001171824 | -2.947931819 |
| C9orf3 | 4.075009 | 16.432379 | 0.01070843 | 2.237319909 |
| PPARA | 4.003935 | 2.116686 | 0.008038534 | -2.328388678 |
| AC010197.2 | 3.624008 | 0.15285 | 5.82E-06 | -4.503971444 |
| VAPB | 3.61449 | 10.800939 | 0.04831702 | 1.691387462 |
| CYB5RL | 3.571304 | 5.420966 | 0.033069804 | 1.84631175 |
| GIPR | 3.355558 | 0.940036 | 0.040876962 | -1.787254404 |
| ZNF707 | 3.306753 | 7.268324 | 0.045626669 | 1.737579026 |
| EGFL8 | 3.237335 | 0.481459 | 0.000765698 | -3.221359686 |
| SCO2 | 3.211192 | 0.002658 | 3.10E-07 | -7.023968695 |
| Human_newGene_3674 | 3.188329 | 0.860956 | 0.037369024 | -1.874832147 |
| FAM156A | 3.184584 | 0.130195 | 9.08E-06 | -4.509177679 |
| DGKH | 3.171043 | 1.976558 | 0.025570055 | -1.931570822 |
| AC106886.5 | 3.053205 | 0.924971 | 0.046360517 | -1.711599263 |
| ZNF641 | 2.9661 | 2.393453 | 0.023180496 | -1.980797414 |
| FKRP | 2.942225 | 7.881124 | 0.001743325 | 2.837863358 |
| Human_newGene_20605 | 2.915903 | 24.310116 | 0.000718593 | 3.062056662 |
| ASB1 | 2.89619 | 10.611487 | 0.00871685 | 2.295130306 |
| ATXN7 | 2.642723 | 0.379421 | 0.000678539 | -3.110252957 |
| EDA | 2.637577 | 0.673559 | 0.019267059 | -2.05741686 |
| ZNF607 | 2.621231 | 0.692868 | 0.034068853 | -1.848845793 |
| LCN2 | 2.606984 | 0.054567 | 4.00E-05 | -4.915386351 |
| AC138696.1 | 2.522276 | 0.446681 | 0.006008695 | -2.475795085 |
| LIPG | 2.463912 | 1.304378 | 0.006213571 | -2.450309014 |
| CLEC11A | 2.319504 | 0.510395 | 0.023047522 | -2.068279949 |
| COMMD6 | 2.300173 | 13.981981 | 0.006210064 | 2.425648976 |
| PDK1 | 2.283959 | 3.10837 | 0.027157829 | 1.912886684 |
| AC020915.1 | 2.253471 | 0.176951 | 0.000127211 | -3.645528396 |
| ZNF587 | 2.206915 | 7.287008 | 0.038222086 | 1.785354954 |
| Human_newGene_7800 | 2.158036 | 0.085526 | 8.65E-05 | -4.533902575 |
| ZNF566 | 2.149321 | 0.500255 | 0.001152504 | -3.031136105 |
| CHST11 | 2.134391 | 4.921373 | 0.024771294 | 1.946161425 |
| GPR20 | 1.951169 | 0.505023 | 0.035559321 | -1.916535439 |
| AC118553.2 | 1.939944 | 0.060213 | 2.11E-05 | -4.303825023 |
| Human_newGene_9930 | 1.937498 | 0.402267 | 0.015448794 | -2.262428855 |
| Human_newGene_8962 | 1.773334 | 0.144753 | 0.00399788 | -3.550913589 |
| AL160269.1 | 1.750164 | 0.293542 | 0.004927142 | -2.558192665 |
| ZNF44 | 1.70346 | 0.370474 | 0.033304866 | -1.906630646 |
| DMAC2L | 1.655894 | 3.501775 | 0.024705085 | -1.965280884 |
| CDKL1 | 1.642059 | 0.403469 | 0.041402174 | -1.849577991 |
| FAM227A | 1.599966 | 1.534004 | 0.024197257 | 1.968850811 |
| THAP9 | 1.556522 | 3.715074 | 0.012999677 | 2.20626606 |
| ZNF573 | 1.548701 | 0.322632 | 0.034303769 | -1.928261022 |
| AC068580.4 | 1.426527 | 0.095812 | 0.003592619 | -2.843153546 |
| Human_newGene_9509 | 1.412942 | 0.111087 | 0.014149938 | -3.122126694 |
| ATXN7 | 1.387973 | 8.167986 | 0.008555 | 2.307520308 |
| AC006064.6 | 1.380542 | 0.000548 | 1.88E-10 | -8.812132311 |
| Human_newGene_3545 | 1.350918 | 0.015278 | 3.40E-06 | -5.847570191 |
| Human_newGene_18091 | 1.348331 | 0.258945 | 0.019841152 | -2.300080413 |
| AC012476.1 | 1.332888 | 0.041758 | 1.95E-05 | -4.935024085 |
| Human_newGene_546 | 1.324226 | 4.998395 | 0.004215864 | 2.52529544 |
| ZNF320 | 1.29831 | 4.000912 | 4.96E-06 | 4.48558292 |
| AMIGO3 | 1.291905 | 0.067971 | 5.97E-05 | -4.137244831 |
| NPB | 1.290584 | 0.206512 | 0.001223496 | -3.212750163 |
| Human_newGene_23929 | 1.272044 | 0.274643 | 0.035233806 | -2.150975724 |
| Human_newGene_9624 | 1.246872 | 0.238125 | 0.038983062 | -2.2147155 |
| NICN1 | 1.244021 | 0.227726 | 0.010852525 | -2.386195536 |
| Human_newGene_7666 | 1.229228 | 0.139834 | 0.017459041 | -2.89879852 |
| ITGB8 | 1.223242 | 3.214258 | 0.007993814 | 2.336007735 |
| Human_newGene_20434 | 1.217006 | 0.080093 | 0.022314819 | -3.242699067 |
| Human_newGene_12166 | 1.206944 | 0.120237 | 0.003598289 | -3.131833712 |
| Human_newGene_14743 | 1.205309 | 4.871456 | 0.019503447 | 2.028136324 |
| LDLRAD2 | 1.205138 | 0.305196 | 0.02974768 | -1.958192891 |
| ZNF91 | 1.190407 | 0.012141 | 3.60E-07 | -6.12366769 |
| AMY2B | 1.181621 | 0.286805 | 0.047581058 | -1.853903548 |
| Human_newGene_17454 | 1.1625 | 0.068464 | 0.002906428 | -3.958790176 |
| Human_newGene_18828 | 1.15251 | 0.189944 | 0.049219036 | -2.448617438 |
| Human_newGene_5531 | 1.15246 | 12.734944 | 2.35E-06 | 4.581300939 |
| CLEC2D | 1.151496 | 0.82579 | 0.02553821 | -2.004253435 |
| Human_newGene_2532 | 1.100739 | 0.131673 | 0.005015008 | -3.013561059 |
| ATAD3C | 1.085989 | 0.171638 | 0.027647815 | -2.049376186 |
| AC024592.3 | 1.081944 | 2.777404 | 0.003885176 | 2.727304205 |
| Human_newGene_12716 | 1.080589 | 0.167385 | 0.046195495 | -2.688550427 |
| Human_newGene_9915 | 1.069313 | 0.271667 | 0.048474468 | -1.958912536 |
| MYL2 | 1.042711 | 3.655604 | 0.046016916 | 1.824317961 |
| Human_newGene_23389 | 1.042695 | 0.087628 | 0.019434951 | -2.991563364 |
| HIST1H3A | 1.036484 | 0.104246 | 0.027322057 | -3.155840004 |
| Human_newGene_9231 | 1.03262 | 4.474204 | 0.016168907 | 2.136070972 |
| ACVRL1 | 1.030723 | 0.011137 | 0.000617763 | -4.149495685 |
| Human_newGene_11174 | 1.028429 | 0.177993 | 0.023136836 | -2.436127926 |
| Human_newGene_3959 | 1.023862 | 0.146963 | 0.03850259 | -2.770475683 |
| Human_newGene_21685 | 1.007704 | 0.2476 | 0.03633082 | -1.960284882 |
| TUBB2B | 0.995299 | 4.251917 | 0.018115129 | 2.103391386 |
| Human_newGene_13701 | 0.984565 | 0.111862 | 0.011706775 | -3.055684568 |
| GOLGA8O | 0.982489 | 0.287579 | 0.021036325 | -2.173948318 |
| Human_newGene_3817 | 0.96386 | 0.175771 | 0.024818572 | -2.294952361 |
| FGF22 | 0.956231 | 0.166771 | 0.015141462 | -2.456806 |
| Human_newGene_14549 | 0.953175 | 0.10691 | 0.017459041 | -2.89879852 |
| Human_newGene_8167 | 0.950447 | 0.236442 | 0.033345889 | -1.988843284 |
| SPARC | 0.930969 | 3.714425 | 0.025212173 | 1.955194217 |
| Human_newGene_16639 | 0.913646 | 0.240691 | 0.044916029 | -1.880221859 |
| Human_newGene_11914 | 0.910207 | 0.051709 | 0.027322057 | -3.155840004 |
| SLPI | 0.900752 | 0.151194 | 0.042028125 | -2.522185382 |
| Human_newGene_17001 | 0.900711 | 0.130082 | 0.013339641 | -2.692809839 |
| Human_newGene_1365 | 0.873578 | 0.079296 | 0.019434951 | -2.991563364 |
| Human_newGene_5697 | 0.871081 | 0.158421 | 0.014824518 | -2.423247004 |
| Human_newGene_17132 | 0.852142 | 0.148981 | 0.020429716 | -2.486551158 |
| Human_newGene_4733 | 0.846194 | 0.038982 | 0.000882847 | -4.320052558 |
| Human_newGene_12596 | 0.837835 | 0.082486 | 0.041756881 | -2.964660192 |
| Human_newGene_17867 | 0.829768 | 0.182378 | 0.036844159 | -2.140885019 |
| UPK3B | 0.818303 | 0.163407 | 0.008252859 | -2.656649587 |
| Human_newGene_2733 | 0.813868 | 0.09514 | 0.015240223 | -2.95301175 |
| HNRNPH2 | 0.809102 | 43.155327 | 2.37E-08 | 5.725915929 |
| Human_newGene_2381 | 0.796947 | 0.0409 | 0.007434817 | -3.612470157 |
| PCED1B | 0.788403 | 0.09261 | 0.027046234 | -2.260282711 |
| Human_newGene_17139 | 0.775736 | 0.064625 | 0.015150304 | -3.402146279 |
| CTAGE9 | 0.770347 | 0.09946 | 0.004632215 | -2.831507535 |
| Human_newGene_16765 | 0.765064 | 0.057007 | 0.012140675 | -3.183229111 |
| WNT2B | 0.753964 | 1.230911 | 0.025916492 | 1.958185108 |
| Human_newGene_15100 | 0.749513 | 2.803182 | 0.028922094 | 1.904088001 |
| Human_newGene_5552 | 0.744463 | 0.050486 | 0.004569388 | -3.795997108 |
| Human_newGene_15010 | 0.742578 | 0.04598 | 0.027322057 | -3.155840004 |
| DLG2 | 0.742423 | 0.018954 | 0.002624146 | -3.241140699 |
| FZD9 | 0.742167 | 0.17569 | 0.029188121 | -2.061877386 |
| KLHDC9 | 0.735442 | 0.133823 | 0.018852638 | -2.429737563 |
| AC008764.4 | 0.695692 | 0.004671 | 0.002525671 | -4.523790959 |
| GAL3ST1 | 0.684133 | 0.248719 | 0.032224685 | -2.188339175 |
| Human_newGene_5410 | 0.680661 | 0.098989 | 0.026707832 | -2.722746493 |
| Human_newGene_20526 | 0.679146 | 0.063229 | 0.041756881 | -2.964660192 |
| Human_newGene_22971 | 0.672876 | 0.022486 | 0.020717199 | -3.769966754 |
| CD33 | 0.646499 | 0.15561 | 0.046377407 | -2.032703072 |
| Human_newGene_3567 | 0.643104 | 0.095463 | 0.015240223 | -2.640560254 |
| COL17A1 | 0.635819 | 0.070264 | 0.036627028 | -1.968939923 |
| OAZ3 | 0.629868 | 1.555115 | 0.028436841 | 2.075274518 |
| ANGPT2 | 0.574145 | 0.116088 | 0.033626782 | -1.982064202 |
| Human_newGene_6363 | 0.572367 | 0.064839 | 0.019434951 | -2.991563364 |
| Human_newGene_141 | 0.565006 | 0.087775 | 0.023136836 | -2.436127926 |
| PRSS33 | 0.563004 | 0.113812 | 0.027591054 | -2.262482452 |
| RIMS2 | 0.553865 | 0.12743 | 0.001659799 | -2.985192796 |
| GJC2 | 0.533448 | 0.118552 | 0.035422422 | -2.10206883 |
| TUSC3 | 0.529878 | 0.128793 | 0.032255894 | -2.010662635 |
| ETFBKMT | 0.512728 | 1.252324 | 0.029333687 | 1.930542833 |
| HIST4H4 | 0.501258 | 0.094602 | 0.014149938 | -3.122126694 |
| ANXA10 | 0.495264 | 1.879462 | 0.034951328 | 1.925216479 |
| BEST4 | 0.493689 | 0.022099 | 0.000696625 | -4.397814049 |
| CAPS | 0.491026 | 0.750109 | 0.003449084 | 2.812883648 |
| ABCF2 | 0.477966 | 0.111816 | 0.043643943 | -2.052588495 |
| CD302 | 0.470063 | 0.022832 | 0.000228024 | -4.260567699 |
| CYP2E1 | 0.469609 | 0.076092 | 0.018523963 | -2.454437899 |
| HYPK | 0.462638 | 2.686227 | 2.92E-05 | 4.081479563 |
| RPRM | 0.458074 | 0.088001 | 0.034088492 | -2.273334858 |
| CYP26A1 | 0.454675 | 0.069996 | 0.01348744 | -2.579552567 |
| FAM24B | 0.452747 | 2.773255 | 0.009611529 | 2.508514147 |
| ZNF550 | 0.43494 | 0.026251 | 0.000326678 | -4.171914868 |
| CHST14 | 0.43241 | 3.462471 | 0.001032703 | 3.006690577 |
| PCDHGB7 | 0.420698 | 0.074129 | 0.003213032 | -2.967258215 |
| SMPD3 | 0.40944 | 1.998162 | 0.000353617 | 3.370018981 |
| CSF2RA | 0.400641 | 0.089714 | 0.036844159 | -2.140885019 |
| GCNA | 0.394875 | 0.041945 | 0.002513798 | -3.180982074 |
| Human_newGene_20459 | 0.394114 | 2.002838 | 0.008440831 | 2.352543815 |
| SNAP25 | 0.392389 | 0.043115 | 0.010299849 | -3.104404824 |
| TAS2R31 | 0.381338 | 0.036289 | 0.033664757 | -3.063414575 |
| NPIPA7 | 0.374261 | 1.746012 | 0.008197228 | 2.516435063 |
| CCT6B | 0.354368 | 1.143117 | 0.029677854 | 2.146926527 |
| TRHDE | 0.350292 | 0.037894 | 0.00089616 | -3.179426089 |
| CHRNB4 | 0.343165 | 1.26883 | 0.040842962 | 1.848906479 |
| ZNF419 | 0.34305 | 0.062592 | 0.046377407 | -2.032703072 |
| Human_newGene_5997 | 0.340867 | 1.753991 | 0.022697535 | 2.330285919 |
| TMEM56-RWDD3 | 0.333755 | 4.563879 | 0.006558623 | 2.592262727 |
| CLCNKB | 0.329998 | 0.032616 | 0.007116069 | -3.197170979 |
| SDR42E2 | 0.32041 | 0.013044 | 0.001904897 | -4.105062229 |
| GCNT3 | 0.312007 | 0.050903 | 0.00132779 | -3.437663988 |
| MINOS1-NBL1 | 0.300859 | 1.241584 | 0.027591054 | 2.359981344 |
| AL049697.1 | 0.297279 | 0.008709 | 0.001280849 | -4.763648164 |
| CAND2 | 0.290031 | 0.048879 | 0.011980945 | -2.545862727 |
| Human_newGene_14586 | 0.287747 | 1.427236 | 0.014694963 | 2.301829618 |
| ZNF772 | 0.28649 | 0.12322 | 0.000478283 | -4.052841796 |
| Z83844.3 | 0.284189 | 0.06146 | 0.040683222 | -2.097963183 |
| RNF112 | 0.270618 | 0.028482 | 0.007116069 | -3.197170979 |
| TUBA8 | 0.26911 | 0.825687 | 0.016730606 | 2.457735696 |
| AD000671.2 | 0.26411 | 0.047256 | 0.049219036 | -2.448617438 |
| Human_newGene_4429 | 0.263641 | 1.02162 | 0.04686269 | 1.946198008 |
| AGMAT | 0.262854 | 0.033801 | 0.020057392 | -2.842468268 |
| AC009336.2 | 0.252841 | 0.050583 | 0.028229227 | -2.265218294 |
| LIPT2 | 0.252662 | 1.363511 | 0.011980945 | 2.625394565 |
| MMP20 | 0.24914 | 0.023385 | 0.018334706 | -3.324624324 |
| Human_newGene_11597 | 0.238682 | 1.42538 | 0.004779776 | 2.583936117 |
| AC022384.1 | 0.234274 | 1.369232 | 0.007862111 | 2.494040995 |
| PCDHGA8 | 0.22749 | 0.007059 | 0.000554409 | -4.471597677 |
| GPR62 | 0.223577 | 0.018789 | 0.018334706 | -3.324624324 |
| MPP4 | 0.222974 | 0.008801 | 0.003029173 | -4.457032807 |
| CFAP52 | 0.213074 | 0.019002 | 0.018334706 | -3.324624324 |
| WRB-SH3BGR | 0.212554 | 1.373661 | 0.032942027 | 2.074038149 |
| HABP2 | 0.212067 | 0.041605 | 0.038983062 | -2.2147155 |
| NPHS1 | 0.21123 | 0.017398 | 0.005623714 | -3.284330886 |
| Human_newGene_131 | 0.210779 | 1.079649 | 0.032224685 | 2.267246531 |
| FBLN2 | 0.20554 | 0.010593 | 0.003112808 | -3.64078925 |
| RBBP8NL | 0.204034 | 0.889918 | 0.023622442 | 2.129773809 |
| AC105052.3 | 0.201026 | 1.136413 | 0.010882272 | 2.485659006 |
| ARHGAP11B | 0.199871 | 3.355061 | 0.000142683 | 3.959923758 |
| TMEM110-MUSTN1 | 0.19428 | 2.403088 | 0.001119686 | 3.507637244 |
| F8A3 | 0.191279 | 1.349829 | 0.004547879 | 2.749181289 |
| MAB21L3 | 0.184969 | 0.027199 | 0.030962986 | -2.658941316 |
| CPED1 | 0.18323 | 0.02918 | 0.004481104 | -3.054072221 |
| Human_newGene_5541 | 0.175448 | 1.078717 | 0.046781564 | 2.318727076 |
| PRSS27 | 0.175337 | 1.146374 | 0.010849845 | 2.597155567 |
| Human_newGene_17844 | 0.174454 | 0.906606 | 0.018577866 | 2.313786936 |
| KCNA7 | 0.174291 | 0.036619 | 0.048450223 | -2.079493812 |
| AL357673.1 | 0.172469 | 0.015659 | 0.022314819 | -3.242699067 |
| ZNF493 | 0.167151 | 0.05956 | 0.020057392 | -2.842468268 |
| NLRP6 | 0.161797 | 0.01558 | 0.03850259 | -2.770475683 |
| AC068946.1 | 0.160531 | 0.017304 | 0.046195495 | -2.688550427 |
| GSTA1 | 0.156681 | 0.924565 | 0.020808432 | 2.544665491 |
| PI15 | 0.156257 | 0.15537 | 0.032224685 | -2.188339175 |
| Human_newGene_2413 | 0.153753 | 1.444403 | 0.009963019 | 2.901527659 |
| IL12RB2 | 0.151534 | 0.010412 | 0.006295194 | -3.676275334 |
| Human_newGene_20474 | 0.151471 | 0.904458 | 0.02310992 | 2.533521738 |
| USP6 | 0.148548 | 0.028662 | 0.021003933 | -2.32105532 |
| NOTCH4 | 0.145627 | 0.023639 | 0.012173415 | -2.618957169 |
| KLRC4-KLRK1 | 0.142566 | 1.264325 | 0.008826286 | 2.662118305 |
| KCTD16 | 0.139853 | 0.184138 | 0.022133394 | -2.154080174 |
| LCT | 0.137442 | 0.005348 | 0.001458763 | -4.15069982 |
| AC090527.2 | 0.136686 | 2.48703 | 0.000201477 | 4.007369115 |
| ALPP | 0.130077 | 0.015396 | 0.033664757 | -3.063414575 |
| ANKFN1 | 0.129537 | 0.011356 | 0.018334706 | -3.324624324 |
| Human_newGene_16077 | 0.12903 | 1.082744 | 0.015240223 | 2.679691203 |
| LILRB3 | 0.124987 | 0.003736 | 0.016225581 | -3.875980995 |
| CD200R1 | 0.12249 | 0.052334 | 0.022314819 | -3.242699067 |
| GJA1 | 0.120646 | 0.517855 | 0.029731472 | 2.07292101 |
| Human_newGene_2599 | 0.118702 | 0.811865 | 0.02310992 | 2.533521738 |
| Human_newGene_9955 | 0.117387 | 0.907067 | 0.00558375 | 2.822281218 |
| NFASC | 0.117053 | 0.500473 | 0.02000023 | 2.091854861 |
| PDE10A | 0.115347 | 0.026417 | 0.038568237 | -2.112618835 |
| FAM47E | 0.112214 | 0.136218 | 0.029884417 | -2.32966511 |
| Human_newGene_23394 | 0.10864 | 0.759018 | 0.033664757 | 2.778473275 |
| Human_newGene_11622 | 0.105265 | 0.974062 | 0.02310992 | 2.533521738 |
| TRIM29 | 0.105026 | 0.425303 | 0.039161722 | 2.004207812 |
| Human_newGene_19923 | 0.101973 | 0.827685 | 0.015240223 | 2.679691203 |
| Human_newGene_10299 | 0.101673 | 0.683566 | 0.042028125 | 2.312285784 |
| Human_newGene_5263 | 0.101597 | 1.047265 | 0.001664863 | 3.323822366 |
| AC131160.1 | 0.101153 | 1.035783 | 0.000871107 | 3.281637127 |
| ZNF615 | 0.101007 | 0.004546 | 0.010215989 | -4.067160807 |
| EPHA8 | 0.099101 | 0.025384 | 0.027120138 | -2.847997639 |
| Human_newGene_928 | 0.098038 | 0.588479 | 0.03850259 | 2.452571312 |
| NPIPA2 | 0.094671 | 0.797787 | 0.010299849 | 2.854060846 |
| KRTAP3-1 | 0.091631 | 1.451318 | 0.001458763 | 3.932625414 |
| Human_newGene_18498 | 0.091323 | 0.972144 | 0.006319303 | 3.00960982 |
| Human_newGene_16921 | 0.090318 | 0.748383 | 0.027120138 | 2.652939458 |
| Human_newGene_7542 | 0.089908 | 1.14115 | 0.000200591 | 3.652265147 |
| AC023055.1 | 0.08924 | 5.9567 | 3.93E-09 | 6.985516096 |
| Human_newGene_1772 | 0.086027 | 0.583267 | 0.018523963 | 2.695742332 |
| HIST2H2BF | 0.083565 | 0.502053 | 0.027591054 | 2.359981344 |
| AC005041.1 | 0.077222 | 0.010502 | 0.042028125 | -2.522185382 |
| KCNQ3 | 0.076807 | 0.44914 | 0.005769578 | 2.53798153 |
| C1orf56 | 0.07639 | 0.629277 | 0.005415811 | 3.009049831 |
| SPTLC3 | 0.070583 | 0.833211 | 0.041406814 | 1.969062589 |
| TMEM256-PLSCR3 | 0.070243 | 6.197689 | 4.94E-06 | 4.907236496 |
| DLEU7 | 0.069699 | 0.369591 | 0.048450223 | 2.240901123 |
| CCDC80 | 0.067857 | 0.292423 | 0.032040594 | 1.961685044 |
| PCDHGA6 | 0.067241 | 0.008096 | 0.041756881 | -2.964660192 |
| HEPH | 0.066869 | 1.035451 | 0.011425695 | 2.398761305 |
| GBP3 | 0.065981 | 0.393529 | 0.035233806 | 2.203308603 |
| Human_newGene_5532 | 0.064903 | 1.510186 | 4.72E-06 | 4.544691614 |
| AC091551.1 | 0.063416 | 0.6554 | 0.004547753 | 3.29625085 |
| Human_newGene_8249 | 0.06341 | 0.494779 | 0.027322057 | 2.860302089 |
| CCDC160 | 0.06051 | 0.378483 | 0.049219036 | 2.251237024 |
| ACTL8 | 0.058299 | 0.304309 | 0.036013059 | 2.370855756 |
| AC008687.8 | 0.055409 | 0.411398 | 0.013339641 | 2.725298856 |
| TNFRSF1B | 0.051724 | 0.529401 | 0.032224685 | 2.267246531 |
| Human_newGene_318 | 0.051688 | 0.786855 | 0.001904897 | 3.854907393 |
| H3F3C | 0.051494 | 0.749094 | 0.00218621 | 3.814419823 |
| ATP12A | 0.050875 | 0.215207 | 0.046377407 | 2.101752742 |
| ARMC12 | 0.047525 | 0.5749 | 0.009030241 | 3.034296998 |
| AL355312.5 | 0.046233 | 1.150878 | 1.16E-05 | 4.56690533 |
| RFLNA | 0.045642 | 0.233337 | 0.036013059 | 2.370855756 |
| Human_newGene_15068 | 0.045153 | 0.576175 | 0.00059879 | 3.617577204 |
| AL031315.1 | 0.043958 | 0.265061 | 0.026707832 | 2.481311391 |
| NRXN2 | 0.040857 | 0.217094 | 0.018244259 | 2.378941522 |
| TEX36 | 0.039872 | 0.398805 | 0.033664757 | 2.778473275 |
| Human_newGene_16650 | 0.039591 | 0.249713 | 0.02310992 | 2.533521738 |
| IL3RA | 0.038017 | 0.25766 | 0.03850259 | 2.452571312 |
| AL139011.2 | 0.036907 | 0.382421 | 0.009963019 | 2.901527659 |
| TSNAX-DISC1 | 0.036642 | 0.209269 | 0.036672434 | 2.411431368 |
| LTA | 0.036117 | 0.30353 | 0.018334706 | 3.011227856 |
| CCDC154 | 0.035525 | 0.326685 | 0.042028125 | 2.312285784 |
| GPR179 | 0.03409 | 0.135573 | 0.048605977 | 2.010624189 |
| AC013717.1 | 0.032006 | 0.273318 | 0.005623714 | 3.046010929 |
| HLA-DQB1 | 0.031389 | 0.23293 | 0.027322057 | 2.860302089 |
| ANKK1 | 0.030964 | 0.242411 | 0.014149938 | 2.883013825 |
| VTCN1 | 0.02956 | 0.302703 | 0.004547753 | 3.29625085 |
| TDRD5 | 0.025748 | 0.225174 | 0.006319303 | 3.00960982 |
| Human_newGene_10709 | 0.02543 | 0.509483 | 0.000997265 | 4.041875125 |
| AC002310.4 | 0.024944 | 1.447887 | 0.00365261 | 4.196487947 |
| CCDC116 | 0.024083 | 0.381388 | 0.001458763 | 3.932625414 |
| RSPH9 | 0.023907 | 0.212821 | 0.019434951 | 2.77255819 |
| BTK | 0.023439 | 0.223007 | 0.012140675 | 2.935224171 |
| CREB3L1 | 0.020256 | 0.292406 | 0.005186874 | 3.25576328 |
| LAMA1 | 0.019036 | 0.091625 | 0.040683222 | 2.219583113 |
| S1PR1 | 0.01871 | 0.125532 | 0.041756881 | 2.691722615 |
| AC012651.1 | 0.018458 | 0.50351 | 0.000108817 | 4.408814854 |
| TMEM249 | 0.018419 | 0.809056 | 0.0006939 | 4.738263008 |
| CRHR2 | 0.017384 | 0.142981 | 0.018334706 | 3.011227856 |
| TMPRSS13 | 0.01665 | 0.110482 | 0.033664757 | 2.778473275 |
| SYN1 | 0.016499 | 0.210935 | 0.007826126 | 3.081393636 |
| STXBP5L | 0.01414 | 0.167837 | 0.001421769 | 3.537411366 |
| MCF2 | 0.013337 | 0.19922 | 0.00218621 | 3.814419823 |
| PAPOLB | 0.012771 | 0.118058 | 0.015150304 | 3.147849332 |
| RPH3A | 0.011284 | 0.097923 | 0.018334706 | 3.081155226 |
| ADH6 | 0.010831 | 0.148721 | 0.022314819 | 2.937737687 |
| ATP8B4 | 0.009412 | 0.11035 | 0.005352624 | 3.493880715 |
| AC008878.2 | 0.008481 | 2.931197 | 9.49E-07 | 6.523887053 |
| CTXND1 | 0.0079 | 0.05289 | 0.041756881 | 2.691722615 |
| AC098650.1 | 0.007314 | 0.125342 | 0.006635587 | 3.935071041 |
| TBX4 | 0.005247 | 0.078692 | 0.045799632 | 3.204557372 |
| DOCK10 | 0.005189 | 0.050439 | 0.033664757 | 2.778473275 |
| CFAP65 | 0.003413 | 0.050509 | 0.045799632 | 3.204557372 |
| Human_newGene_20757 | 0.001543 | 0.750799 | 1.51E-07 | 6.988283938 |
| AC012531.3 | 0.001472 | 0.433001 | 0.020717199 | 3.523263111 |
| AC020915.5 | 0.000744 | 0.23104 | 0.001092649 | 4.563893365 |

**Table S6.** Primer Sequences

| **Primer name** | **Primer sequence** |  |
| --- | --- | --- |
| ChIP-F1 | TGTGGATCTGAATTGTCAGT | ChIP Primers |
| ChIP-R1 | GGAATACCATGATGCTGTTG |  |
| ChIP-F2 | TGTTCTCCGCCAAATGAGTG |  |
| ChIP-R2 | GGTATTTGAAGTTATGGGGCTTCT |  |
| ChIP-F3 | TGAGTAAGGCAAATGCCAAA |  |
| ChIP-R3 | CCTATAAAGTGCCACTCATCAC |  |
| ChIP-F4 | GATAGAGTGAGCTCTTGTCC |  |
| ChIP-R4 | ACCAAAACTCATGTTGAACC |  |
| Promoter-ChIP-F | AGGAATGCAGAGGTCTAC |  |
| Promoter-ChIP-R | ACTTACTTTCCCACACAAC |  |
| E1-F | TCCTATCCTCTACCTTCCCA | Primers for dual luciferase reporter |
| E1-R | AGATCCCAGACTACTGCTAGA |  |
| E2-F | TGTTGATGACCCACCAGTGT |  |
| E2-R | CCCATAAAGTGCCAACAACAAC |  |
| E3-F | GTGCTTTGAGAACATGGCAA |  |
| E3-R | CCTGCCATACATCATTCCACA |  |
| E4-F | GGGGTCTTACATCCCTTCTC |  |
| E4-R | CAGAGGAGTTTGTTGTCTTCAC |  |
| E3-1-F | CCGCTCGAGGTGCTTTGAGAACATGG |  |
| E3-1-R | CGGGGTACCTGTTGGAAATCTTGCCT |  |
| E3-2-F | CCGCTCGAGGTGAGGCAAGATTTCCA |  |
| E3-2-R | CGGGGTACCATGTCAAGAA |  |
| E3-3-F | CCGCTCGAGATGAGACAGAGGTTCTT |  |
| E3-3-R | CGGGGTACCCCTCATATACAAAGTCC |  |
| E3-4-F | CCGCTCGAGTTGGGGACTTTGTATAT |  |
| E3-4-R | CGGGGTACCCCATACATCATTCCACA |  |
| E3-2-1-F | CCGCTCGAGGTGAGGCAAGATTTCCA |  |
| E3-2-1-R | CGGGGTACCAATAATAGTCTGTATGATTC |  |
| E3-2-2-F | CCGCTCGAGCTTACTTTGATCCTTTCATC |  |
| E3-2-2-R | CGGGGTACCATGTCAAGAACCTCTGT |  |
| E3-2-3-F | CCGCTCGAGAACACTAAAAATGATTA |  |
| E3-2-3-R | CGGGGTACCAGATGAAAGGATCAAAG |  |
| del1-F | CCGCTCGAGAACACTAAAAATGAT |  |
| del1-R | CGGGGTACCATCATTTTTAGTGTT |  |
| del2-F | CCGCTCGAGATGAGTCAT |  |
| del2-R | CGGGGTACCATGACTCAT |  |
| del3-F | CCGCTCGAGCCAGGAAGTT |  |
| del3-R | CGGGGTACCAACTTCCTGG |  |
| del4-F | CCGCTCGAGAGATGACACATTT |  |
| del4-R | CGGGGTACCAAATGTGTCATCT |  |
| E3-2-gRNA1-F | CACCGGAAGAACTTACTAGTGATCTA | sgRNAs for CRISPR/Cas9 |
| E3-2-gRNA1-R | AAACTAGATCACTAGTAAGTTCTTCC |  |
| E3-2-gRNA2-F | CCGGGTAAATCACCCTAACTTCCT |  |
| E3-2-gRNA2-R | AAACAGGAAGTTAGGGTGATTTAC |  |
| del hot spot 2-gRNA1-F | CCGGATTGGCATGTAATTTAGAGACCT |  |
| del hot spot 2-gRNA1-R | TCTCTAAATTACATGCCAATCAAA |  |
| del hot spot 2-gRNA2-F | CACCGGTAAATCACCCTAACTTCCTG |  |
| del hot spot 2-gRNA2-R | CCCAGGAAGTTAGGGTGATTTACAAA |  |
| DCBLD2-F | CTCCTGCTCTTACTTGTCCT | Primers for qRT-PCR. |
| DCBLD2-R | CATTCACAAACAGTGCTGTTG |  |
| GAPDH-F | ATGGGGAAGGTGAAGGTCG |  |
| GAPDH-R | GGGGTCATTGATGGCAACAATA |  |
| Fos-F | AAGATGGCTGCAGCCAAATGCC |  |
| Fos-R | GGTTGGCAATCTCGGTCTGCAAAG |  |
| JunB-F | AGACGCTCAAGGCCGAGAAC |  |
| JunB-R | TGTCCCTTGACCCCAAGCAG |  |
| FOSL2-F | TGTCCCTTGACCCCAAGCAG |  |
| FOSL2-R | TGTCCCTTGACCCCAAGCAG |  |
| JunD-F | GAAGACCCTCAAGAGTCAGAACAC |  |
| JunD-R | GTTGACGTGGCTGAGGACTT |  |
| VEGF-F | TCCGCAGACGTGTAAATGTTCC |  |
| VEGF-R | GCGTGGTTTCTGTATCGATCGT |  |
| TNFRSF6B-F | GAAACACCCACCTACCCCTG |  |
| TNFRSF6B-R | TCCAGAACTGCGTGTAGTGG |  |
| PI3K-F | GGACCCGATGCGGTTAGAG |  |
| PI3K-R | ATCAAGTGGATGCCCCACAG |  |
| CD146-F | AGCTCCGCGTCTACAAAGC |  |
| CD146-R | CTACACAGGTAGCGACCTCC |  |
| P-AKT-F | TACGAGATGATGTGCGGTCG |  |
| P-AKT-R | GGAAGCGGATCTCCTCCATG |  |
| β-Actin-F | GTTGCTATCCAGGCTGTG |  |
| β-Actin-R | TGATCTTGATCTTCATTGTG |  |
| siFos-F | GGAGACAGACCAACUAGAAGA | siRNA sequences |
| SiFos-R | UUCUAGUUGGUCUGUCUCCGC |  |
| SiJunB-F | CAUCAACAUGGAAGACCAAGA |  |
| SiJunB-R | UUGGUCUUCCAUGUUGAUGGG |  |
| SiJunD-F | CGAGCUCACAGUUCCUCUACC |  |
| SiJunD-R | UAGAGGAACUGUGAGCUCGUC |  |
| SiFOSL2-F | GGCCCAGUGUGCAAGAUUAGC |  |
| SiFOSL2-R | UAAUCUUGCACACUGGGCCGU |  |
| DCBLD2-Biotin-F | GTGAGGCAAGATTTCCAAC | Primers labeled with biotin probes |
| DCBLD2-Biotin-R | ATGTCAAGAACCTCTGTCTCAT |  |
| hot spot 1-F | TATCCTAACAATATACCTTC | E3-2 component mutation primers |
| hot spot 1-R | GGAAATCTTGCCTCACCTCG |  |
| hot spot 2-F | ACAGACTATTATTCTTACTT |  |
| hot spot 2-R | TGGCATGTAATTTAGAGAAG |  |
| hot spot 3-F | AGGGTGATTTACTAGATGAC |  |
| hot spot 3-R | GGAGATGAAAGGATCAAAGT |  |
| hot spot 4-F | CCTAATGGTTATGAGACAGA |  |
| hot spot 4-R | AGTAAATCACCCTAACTTCC |  |

**Table S7.** ChIP-seq is used to identify and analyze super-enhancers

| Cell Type | Histone Marker / TF | GEO or ENCODE |
| --- | --- | --- |
| HCT-116 | H3K27ac | GSM2534278 |
| SW480 |  | GSM2746538 |
| SW620 |  | GSM2532779 |
| HMEC |  | **GSE118793** |
| NHEK |  | **GSE90770** |
| HUVEC |  | **GSE136661** |
| A549 |  | GSM2406904 |
| GM12878 |  | **GSE31477** |
| H1-hESC |  | **GSE36133** |
| K562 |  | **GSE31477** |
| HELA-S3 |  | **GSE113674** |
| HSMMTUBE |  | **GSE71452** |
| PANC1 |  | GSM2466034 |
| NCM460 |  | **GSE113674** |
| HCT-116 | H3K4me1 | [GSM2711385](http://www.ncbi.nlm.nih.gov/geo/query/acc.cgi?acc=GSM2711385) |
| NCM460 |  | GSE113674 |
| HCT-116 | DNase | GSM1224667 |
| NCM460 |  | **GSE151464** |
| HCT-116 | P300 | GSM1240110 |
| NCM460 |  | **GSE113674** |
| HCT-116 | ATAC-seq | **[ENCSR357OXA](https://www.encodeproject.org/experiments/ENCSR357OXA/)** |

**Supplementary Fig. S1 to Fig. S7**

**Fig. S1. a** ImageJ analyzed the relative protein expression levels of DCBLD2 in HCT116 and SW480 cells treated with different concentrations of JQ1 (0-5000 nM for 24 h in Fig. 2h. The expression of GAPDH was used to standardize the Western blot results (n=3). **b** ImageJ analysis of Fig. 2i showing the relative protein expression levels of DCBLD2 in HCT116 and SW480 cells after time-dependent treatment with 500 nM JQ1 (0-24 h). The expression of GAPDH was used to standardize the Western blot results (n=3). **c** RT-qPCR was used to detect the relative mRNA expression of *DCBLD2*, *ALB*, and *GUSB* in HCT116 and SW480 cells treated with 500 nM JQ1 for 24 h. *ALB* and *GUSB* as negative control genes regulated by non SEs; DMSO treatment as solvent control. The expression of *GAPDH* was used to standardize the RT-qPCR results (n=3). **(a-c)** Three biological replicates' means are displayed. SEMs are indicated by error bars. With a two-tailed Student's t test, **P* < 0.05, ***P* < 0.01, ****P* < 0.001, and ns, not significant. **d** Genome browser (https://guolab.wchscu.cn/hTFtarget) tracks show TFs binding density signals at the SE3 site of DCBLD2-SE in the HCT116 and SW480 cell lines.

**Fig. S2. a** Schematic DNA sequencing alignment of four AP-1 hot spot deletion mutants (del hot spot 1-4) within the E3-2 core enhancer fragment. **b** JASPAR (https://jaspar.genereg.net/) transcription factor binding motif prediction of the SE3-2 region, listing all AP-1 family TFs capable of recognizing the hot spot 2 core sequence. **c** RT-qPCR analysis detecting relative mRNA expression of Fos, JunB, JunD, Fosl2 and Jun after transfection of corresponding siRNAs in HCT116 cells (siNC, negative control siRNA; n=3). The expression of *GAPDH* was used to standardize the RT-qPCR results. Three biological replicates' means are displayed. SEMs are indicated by error bars. With a two-tailed Student's t test, ***P* < 0.01, ****P* ﹤ 0.001. **d** Silver staining image of biotinylated SE3-2 DNA pull-down products from HCT116 nuclear extracts. Input: total nuclear protein input control; SE3-2: unbiotinylated SE3-2 probe negative control; SE3-2-Biotin: biotin-labeled SE3-2 probe pull-down group; protein molecular weight marker (kDa) is shown on the left.

**Fig. S3. a** ImageJ quantitative analysis of DCBLD2 protein expression levels in HCT116, SW480, and LoVo cells for the WT and DCBLD2-SE loss-of-function mutants (SE#1^-/-^ and SE#2^-/-^) shown in Fig. 5d. The expression of GAPDH was used to standardize the Western blot results (n=3). Three biological replicates' means are displayed. SEMs are indicated by error bars. With a two-tailed Student's t test, ***P* < 0.01, ****P* < 0.001. **b** Immunofluorescence staining for DCBLD2 (green) and DAPI (blue) in SW480 and LoVo cells. Scale bar, 10 μm. **c** The Hi-C sequencing heatmap shows DEGs associated with abnormal A/B compartment transitions, TAD interaction strength, and chromatin loop formation between HCT116 WT and SE#1^-/-^ cells. The color gradient represents the standardized fold change in gene expression (red, upregulated; blue, downregulated).

**Fig. S4. a** Hi-C analysis of KEGG pathway enrichment for DEGs in the TAD region of HCT116 cells (WT and SE#1^-/-^). The x-axis represents the number of enriched genes in each pathway, the y-axis lists the pathway names, and color intensity corresponds to -log10(*P*-value). **b** TFs binding motifs with differing ATAC-seq peak enrichment between WT and SE#1^-/-^ cells in HCT116. **c** RNA seq analysis revealed functional enrichment of DEGs GO in SE#1^-/-^ cells of HCT116. **d** ImageJ analysis of relative protein expression levels of DCBLD2 in the rescue group of HCT116, SW480, and LoVo cells overexpressing DCBLD2 in Fig. S6f. The expression of GAPDH was used to standardize the Western blot results (n=3). Three biological replicates' means are displayed. SEMs are indicated by error bars. With a two-tailed Student's t test, **P* < 0.05, ***P* < 0.01, and ns, not significant.

**Fig. S5. a** RT-qPCR analysis detecting relative mRNA expression of DCBLD2 after transfection of corresponding siRNAs in HCT116, SW480, and LoVo cells (siNC, negative control siRNA; n=3). The expression of *GAPDH* was used to standardize the RT-qPCR results. **b** Western blot and imageJ quantification results of DCBLD2 protein in siNC and siDCBLD2 groups of three CRC cell lines. The expression of GAPDH was used to standardize the Western blot results (n=3). **c** RT-qPCR detection of mRNA levels for downstream genes after DCBLD2 inhibition in HCT116, SW480 and LoVo cells (siNC, negative control siRNA; n=3). The expression of *GAPDH* was used to standardize the RT-qPCR results. **d** Quantitative analysis using ImageJ of the relative protein expression levels of downstream genes in HCT116, SW480, and LoVo cell lines shown in Fig. S6h. The expression of GAPDH was used to standardize the Western blot results (n=3). **e** Western blot and imageJ quantification results of downstream pathway proteins in the siNC and siDCBLD2 groups of HCT116, SW480, and LoVo cell lines. GAPDH expression was used to standardize Western blot results (siNC, negative control siRNA; n=3). **f** Quantitative analysis of relative protein expression levels of DCBLD2 after SR11302 inhibitor action on HCT116 SW480 and LoVo in Fig. 7b using imageJ. The expression of GAPDH was used to standardize the Western blot results (n=3). **(a-f)** Three biological replicates' means are displayed. SEMs are indicated by error bars. With a two-tailed Student's t test, **P* < 0.05, ***P* < 0.01, ****P* < 0.001 and ns, not significant.

**Fig. S6. a** Wound healing assays were conducted on SW480 and LoVo cells at 0 and 48 h to assess their migratory capacity, and the relative migratory capacity was quantified (scale bar, 100 μm; n=3). **b and c** The Transwell assay was used to assess the invasive and migratory capabilities of SW480 and LoVo cells. Quantitative analyses of migration and invasion are shown in the figure on the right (scale bar, 100 μm; n=3). **d** The colony formation assay was conducted to evaluate the long-term proliferation capacity of SW480 and LoVo, and the number of colonies was counted and statistically analyzed (n=3). **(a-d)** Three biological replicates' means are displayed. SEMs are indicated by error bars. With a two-tailed Student's t test, **P* < 0.05, ***P* < 0.01 and ****P* < 0.001.

**Fig. S7. a** Representative photographs of subcutaneous xenograft tumors in nude mice from four groups: the HCT116 WT and SE#1^‑/‑^ groups, as well as the 0.5 mg/kg/day SR11302 treatment group and the DMSO control group (n=5). SR11302 was administered via intraperitoneal injection. All tumor images were captured against the same background and with consistent scale bar settings. **b** ImageJ was used to analyze and quantify the proteins in the Western blot samples from the xenograft tumor tissues of the four experimental groups shown in Fig. 8e. GAPDH expression was used to standardize Western blot results. Three biological replicates' means are displayed. SEMs are indicated by error bars. With a two-tailed Student's t test, ***P* < 0.01 and ****P* < 0.001. **c** Representative images of organ tissues isolated from the HCT116+DMSO and HCT116+SR11302 treatment groups, with a standard ruler included for size comparison. **d** Hematoxylin-eosin stained sections of organ tissues from nude mice in the HCT116+DMSO and HCT116+SR11302 groups (scale bar, 100 μm).
